# How Peroxisomes Affect Aflatoxin Biosynthesis in *Aspergillus Flavus*

**DOI:** 10.1371/journal.pone.0048097

**Published:** 2012-10-19

**Authors:** Massimo Reverberi, Marta Punelli, Carrie A. Smith, Slaven Zjalic, Marzia Scarpari, Valeria Scala, Giorgia Cardinali, Nicaela Aspite, Flavia Pinzari, Gary A. Payne, Anna A. Fabbri, Corrado Fanelli

**Affiliations:** 1 Dipartimento di Biologia Ambientale, Università Sapienza, Roma, Italy; 2 Oklahoma State University, Oklahoma City, Oklahoma, United States of America; 3 Department of Ecology Agronomy and Aquaculture, University of Zadar, Zadar, Croatia; 4 IFO-S.Gallicano, Roma, Italy; 5 IRCPAL, Ministero dei Beni Culturali, Roma, Italy; 6 North Carolina State University, Raleigh, North Carolina, United States of America; Soonchunhyang University, Republic of Korea,

## Abstract

In filamentous fungi, peroxisomes are crucial for the primary metabolism and play a pivotal role in the formation of some secondary metabolites. Further, peroxisomes are important site for fatty acids β-oxidation, the formation of reactive oxygen species and for their scavenging through a complex of antioxidant activities. Oxidative stress is involved in different metabolic events in all organisms and it occurs during oxidative processes within the cell, including peroxisomal β-oxidation of fatty acids. In Aspergillus flavus, an unbalance towards an hyper-oxidant status into the cell is a prerequisite for the onset of aflatoxin biosynthesis. In our preliminary results, the use of bezafibrate, inducer of both peroxisomal β-oxidation and peroxisome proliferation in mammals, significantly enhanced the expression of pex11 and foxA and stimulated aflatoxin synthesis in A. flavus. This suggests the existence of a correlation among peroxisome proliferation, fatty acids β-oxidation and aflatoxin biosynthesis. To investigate this correlation, A. flavus was transformed with a vector containing P33, a gene from Cymbidium ringspot virus able to induce peroxisome proliferation, under the control of the promoter of the Cu,Zn-sod gene of A. flavus. This transcriptional control closely relates the onset of the antioxidant response to ROS increase, with the proliferation of peroxisomes in A. flavus. The AfP33 transformant strain show an up-regulation of lipid metabolism and an higher content of both intracellular ROS and some oxylipins. The combined presence of a higher amount of substrates (fatty acids-derived), an hyper-oxidant cell environment and of hormone-like signals (oxylipins) enhances the synthesis of aflatoxins in the AfP33 strain. The results obtained demonstrated a close link between peroxisome metabolism and aflatoxin synthesis.

## Introduction

Peroxisomes are ubiquitous organelles with an oxidative-type metabolism which fulfils a range of metabolic functions [1], [2]. In fungi, peroxisomes are involved in the β-oxidation (together with mitochondria) of long-chain fatty acids [3], oxalate synthesis and the metabolism of methanol, amines and alkanes, in antibiotics synthesis [4] and probably in the first steps of mycotoxin synthesis [5]. Many authors have recently focalized their attention on the role of peroxisomes and the genes responsible for their proliferation (e.g. *pex11*) and for the regulation of fatty acids metabolism by β-oxidation (such as *foxA*)[5], [6]. Among these functions, beside the β-oxidation of long chain fatty acids (oleic, linoleic fatty acids and others) [7], peroxisomes are engaged in the metabolism of reactive oxygen species (ROS). In fact, several studies indicate that the peroxisomes are crucial knots in the metabolism of ROS, reactive nitrogen species (RNS) and in β-oxidation with concomitant production of intra- and inter-cellular signalling molecules. Since these molecules are produced during normal cell metabolism, their role in signalling largely depends on the balance between synthesis, utilisation and degradation [7], [8], playing a key role in redox-regulated responses. Thus, peroxisomes play an important role in the regulation of cell redox balance and, in relation to this, they are involved in different signal transduction pathways [9] correlated to the cell response towards oxidative stress. These stressful conditions are originated, for instance, when toxigenic fungi try to colonise their hosts, e.g. maize seeds [10]. In these conditions, peroxisomes are prompted to proliferate in *A. nidulans* when the fungus is grown onto viable maize seeds [5]. Some secondary metabolites are synthesised by fungi during morphological (e.g sexual or asexual reproduction) and metabolic transitions (idiophase/trophophase), which are events starting when ROS accumulation occurs [11]. The creation of an oxidative intracellular environment, that is the oxidative unbalance, trigger AF biosynthesis [12], [13]. The relationship between oxidative stress and AF synthesis has been further corroborated by the finding of the presence in *A. parasiticus* cell of the oxidative-stress transcription factor Ap*yapA* which “senses” the oxidative stress into the cytoplasm and, by transcriptional control together with AftB, activates, ultimately, antioxidant enzymes to scavenge the excess of oxidants [13], [14]. In fact, the reduction of the cell environment leads to the control/inhibition of AF synthesis [15]. Up to day the knowledge concerning the role played by peroxisomes and AF biosynthesis clearly suggests their involvement in toxin synthesis even if it is not clear the cascade of events by which peroxisome proliferation supports and enhances AF biosynthesis [5].

Here we present a report which investigates the correlation amongst peroxisomes proliferation, ROS/oxylipins production and AF biosynthesis in *A. flavus*. At the beginning, bezafibrate a drug known in animal studies with positive effect on peroxisome proliferation *via* PPARα receptor [16], was used in *A. flavus* to indirectly demonstrate an eventual relationship between aflatoxin synthesis and peroxisome proliferation. Subsequently, with the aim to more directly demonstrate this correlation, this fungus was transformed with the gene encoding for the protein P33 of the *Cymbidium ringspot* virus, which triggers peroxisome proliferation in plant and in *Saccharomyces cerevisiae* [17]–[19] and whose expression is controlled by the promoter of AFLA 099000, a Cu,Zn-superoxide dismutase. In this way we try to link the onset of antioxidant response to ROS accumulation with the peroxisome proliferation in *A. flavus*. This study assigns to peroxisomes a significant role in the cascade of events correlated to AF biosynthesis.

## Results

### Mammals peroxisome proliferator enhances pex11 and foxA expression and triggers aflatoxin biosynthesis in A. flavus

The addition of BEZAfibrate (BEZA) an enhancer of both peroxisomal β-oxidation and peroxisome proliferation [16], to an AF-low conducive media (CD) promoted pex11 and foxA gene expression (∼9 and 13 folds respectively compared to untreated samples) and stimulated AF biosynthesis (1.1±0.1 μg/mL in CD+BEZA vs 0.11±0.005 μg/mL in CD) without affecting fungal growth (4.96 mg/mL ±0.55 in CD+BEZA vs 5.02 mg/mL ±0.41 in CD). This result suggested the existence of a correlation between peroxisome proliferation and AF biosynthesis modulation (Table 1).

**Table 1:** Aflatoxin (µg/mL) synthesis, fungal growth (mg/mL d.w.), *pex11* and *foxA* mRNA relative expression in *A. flavus* 3357 (WT) grown in basal media (CD) for aflatoxin (AF) synthesis after 7 days at 30°C.

	Fungal growth (mg/mL d.w.)	AF (µg/mL)	*pex11* mRNA rel. Expression	*foxA*mRNA rel. expression
**Control (LCM)**	5.02±0.41	0.11±0.005	-	-
**BEZA 1mM**	4.96±0.55	1.10±0.120	9.2	13.0

### Construction and characterisation of A. flavus strains expressing the P33 gene

**Figure 1:**
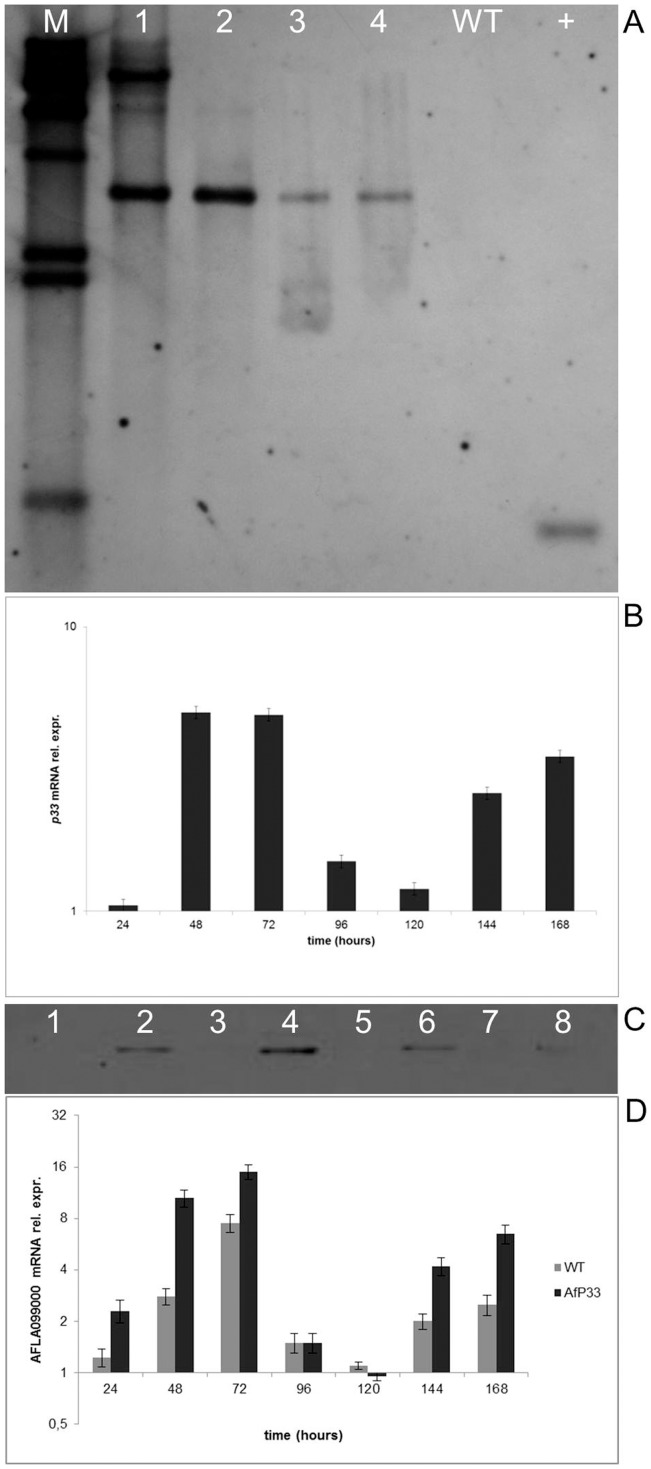
Molecular analysis of AfP33 transformants. (A) Southern analysis of NRRL 3357 strain (WT) and AfP33 putative transformants (1–4). Genomic DNA was digested with EcoRI to determine p33 insertion as single copy. A 0.5 kb PCR product contained in p33 gene (also used as positive control, +) was used as a probe. The hybridization temperature was at 65°C. λ_hindIII_ (Roche, Swiss) was used as molecular weight marker (M). (B) Real Time PCR analysis of p33 mRNA expression in A. flavus AfP33 strain relative to the time 0 (inoculation), grown in CD after different periods of incubation (0 up to 168 hpi). (C) Western blot analysis of total proteins of A. flavus (wild-type strain NRRL 3357 -WT- and AfP33 transformant – T) by using an anti-p33 anti-serum kindly provided by Dr. L. Rubino. P33 expression was analyzed after 48, 72, 96 and 168h of incubation in CD medium at 30°C in the WT (lanes 1-3-5-7) and AfP33 (lanes 2-4-6-8) strains. Pure P33 was used for comparison. (D) AFLA099000 (Cu,Zn-sod) mRNA expression in the WT and AfP33 strains after 24, 48, 72, 96, 120, 144 and 168h of incubation in CD medium at 30°C normalized to the time 0 (inoculation time).

Maggio-Hall et al, [5] clearly indicated that the peroxisomal β-oxidation is a fundamental step for aflatoxin biosynthesis. The use of peroxisome proliferators (see above) confirmed the role of peroxisome and β-oxidation into the aflatoxin metabolism. Thus, as suggested by several papers [17]–[19], we individuated the viral protein P33, which induces peroxisome proliferation in plant and yeast and, for the first time at our knowledge, we have tried its effect on the peroxisomes of filamentous fungi by inserting the P33 gene into A. flavus. Moreover, the expression of the P33 gene of Cymbidium ringspot virus was regulated by the promoter region of the Cu,Zn-sod gene (AFLA099000) of A. flavus. This is also the first report in which this promoter was used for controlling the expression of an exogenous gene such as P33 in A. flavus. The analysis of the regulatory elements present in the 2.0 Kb 5' Flanking sequence (upstream) of AFLA099000, retrieved from http://fungi.ensembl.org/Aspergillus_flavus/Info/Index, was performed with the genomic tools in the aspergillusflavus.org website and through the NSITE tool in the softberry.com website and the results are shown in supplementary materials (Table S1). The promoter presented several regulatory motifs amongst which a responsive element for AP-1, the master regulator of antioxidant response in filamentous fungi [13], [20]. Thus, we hypothesized that the expression of AFLA099000 could be regulated by oxidative stress onset into A. flavus cell, which, as shown below (Figure 1D) presented an early (24–48 h) and a late (144–168 h) peak of accumulation.

AfP33 and WT strains were analysed by Southern, Western blots and Real Time PCR. As expected, after EcoRI restriction of WT and AfP33 DNA, one fragment at ∼4.4 Kb hybridized to the P33 probe (Figure 1A). The expected hybridization band patterns for EcoRI digestion were a single band for the transformant strain. Southern blot analysis indicated that the P33 gene sequence was inserted in one copy in the transformants 2–4 (two in the transformant strain#1) and it was absent in the WT strain. The Real Time PCR analysis showed that the P33 mRNA was expressed, as expected, in the sole AfP33, with expression peaks at 48–72 and 144–168 hours post inoculation (hpi) (Figure 1B). The Western blot analysis confirmed that P33 protein is expressed by the sole AfP33 strain and, in the time intervals tested, its expression is closely related to P33 mRNA expression (Figure 1C). The expression of AFLA099000, whose promoter was used for controlling P33 expression was monitored too. As expected, its expression, even if with slightly different levels, showed a trend similar to P33 in both WT and AfP33 strains (Fig. 1D). As shown in Figure S1, apart one transformant (the #1 in Fig. 1) which shows an altered profile respect to #2, the other transformant strains behave similarly to #2 concerning P33, foxA, pex11 mRNA expression and AFB1 biosynthesis.

The effect of P33 on peroxisomes probably affects other aspects of the metabolism as shown by the data obtained by a phenotypic microarray based approach (Table S2) in which different pathways of fungal metabolism resulted significantly affected in the AfP33 strain in comparison with the WT strain. The pathways comparison indicated that glyoxylate and dicarboxylate metabolism (1,17±0,02 *vs* 1,11±0,01 Abs_450_; p<0,001), propanoate metabolism (1,16±0,02 *vs* 1,12±0,02 Abs_450_; p<0,01) and the TCA cycle (1,13±0,02 *vs* 1,09±0,02 Abs_450_; p<0,01) were significantly up-regulated in AfP33 in comparison with WT strain; albeit, glycolysis/gluconeogenesis (1,31±0,02 *vs* 1,35±0,02 p<0,05) and sucrose metabolism (1,30±0,01 *vs* 1,35±0,01 Abs_450_; p<0,01) were down-regulated in AfP33 (Table S2).

### AfP33 ultrastructural characterization

**Figure 2:**
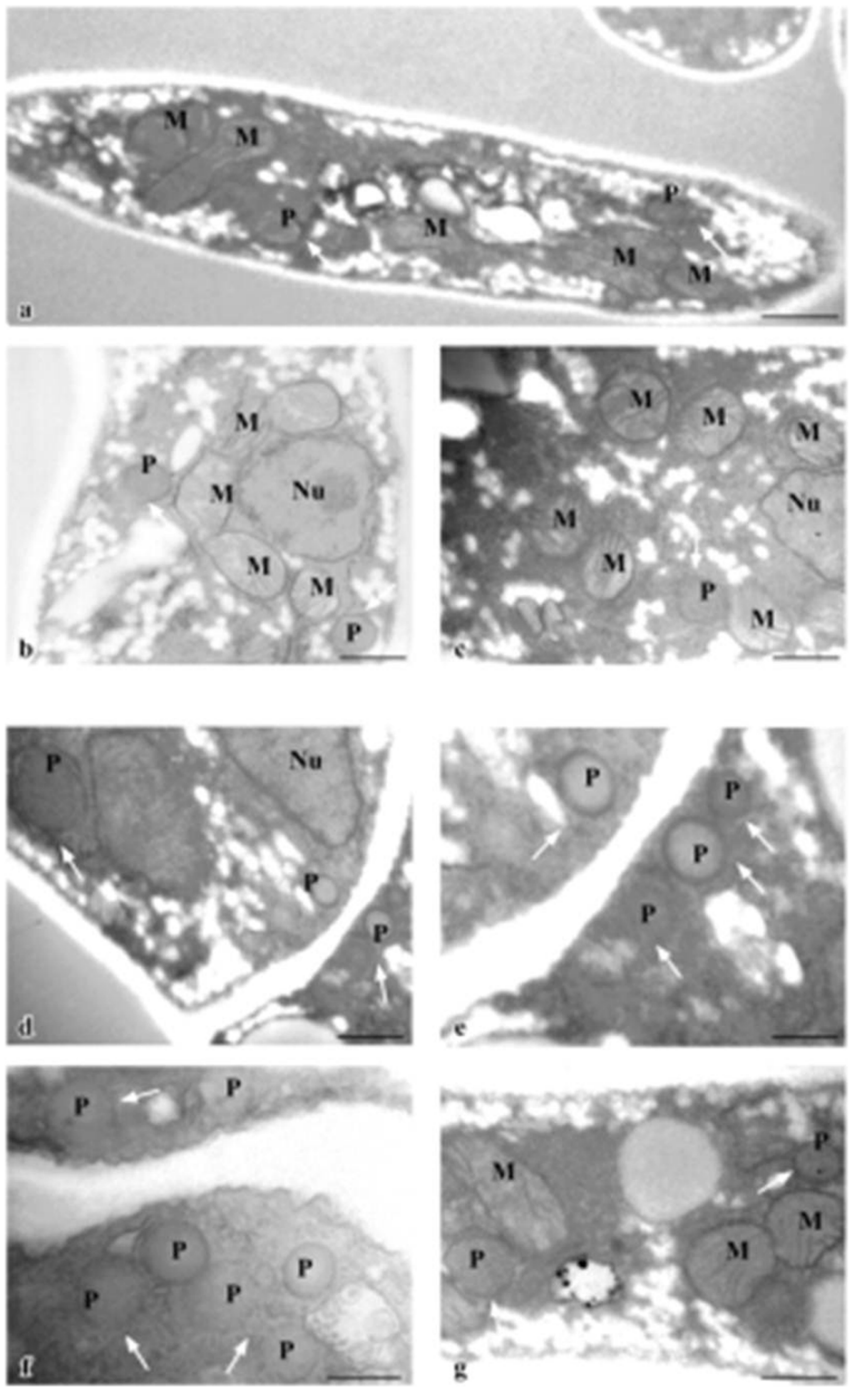
Ultrastructural characterization. TEM examination of A. flavus mycelia. Expression of P33 in AfP33 cells (D, E, F, G) significantly increases number of peroxisomes in comparison with WT cells (A, B, C). Bar 0.5 μm in A, B, C, D; bar 0.25 μm in E; bar 0.5 μm in F, G. (P, Peroxisomes; M, mitochondria; Nu, nucleus).

Transmission electron microscopic (TEM) analysis was carried out to investigate if the P33 protein expression induced peroxisomes proliferation in A. flavus as was previously demonstrated in yeast [18]. AfP33 cells contained a higher number of organelles, which were identified by TEM as peroxisomes, in comparison with the WT (Figure 2A–G). Ultrastructural analysis confirmed that P33 expression is correlated with an increase in the number of peroxisomes. In fact, AfP33 (Figure 2D–G), showed a cytoplasm which is more enriched in membranous organelles, identified as normal-sized peroxisomes (P) sometimes mixed with mitochondria (M), in comparison with the WT (Figure 2A–C). We quantified the amount of peroxisomes in AfP33 respect to WT by counting the number of organelles/cell section considering for each sample at least 50 cells, randomly taken. The analysis showed a significant increase of the peroxisome number in AfP33 compared to WT (3.85±1.3 and 2.6±0.7 respectively; p<0.01). Results are expressed as the mean number of peroxisomes/cell section ± SD.

### AfP33 fluorescence microscopy characterization

**Figure 3:**
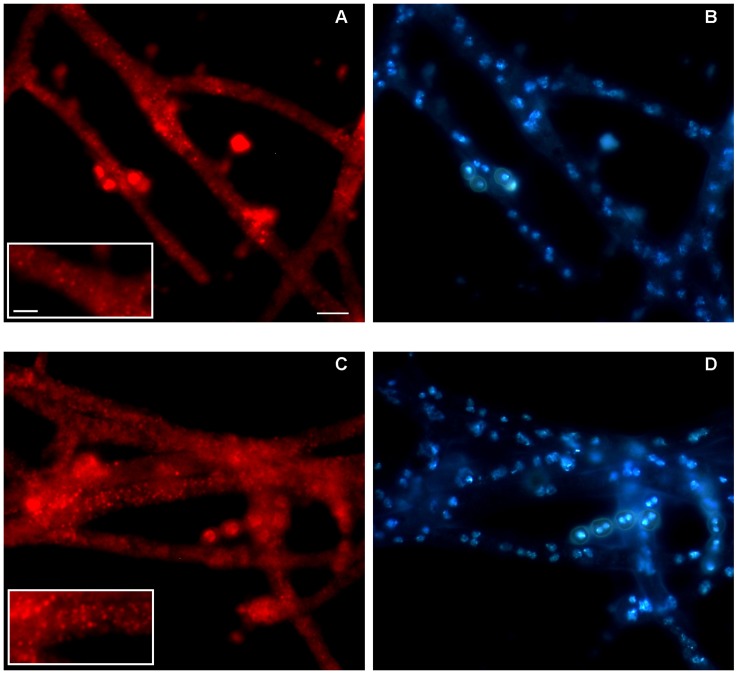
Fluorescence analysis of AFDsRED and AfP33 strains. The fluorescence analysis was performed to compare the presence of peroxisomes (red) between (A, B) AfDsRED and (C, D) AfP33 strains. Cells not expressing p33 displayed a punctate pattern of fluorescence (A and detail in the boxed area) corresponding to scattered peroxisomes whereas cells expressing p33 showed the presence of red spots revealing an increased number of peroxisomes (C and detail in the boxed area). Nuclei are stained with DAPI. Scale bars =  10 mm (A–D); 5 mm (boxed areas in A and C).

Af-DsRED strain transformed with the plasmid pSDsRED, which contained the coding sequence of the red fluorescent protein DsRED with the PTS1 (peroxisomal targeting signal, -SKL), showed a punctate cytoplasmic pattern of red staining which identified the presence of few small scattered peroxisomes (Figure 3A–B). AfP33 cells showed a significant over-representation of the red fluorescent spots (Figure 3C–D), which indicated the presence of peroxisome proliferation induced by P33 from a mean of 7.25±0.25 peroxisome/μm^2^ (WT) to 10.5±1.5 peroxisome/μm^2^ (AfP33) (P<0.001).

### Fungal growth and conidiogenesis

**Figure 4:**
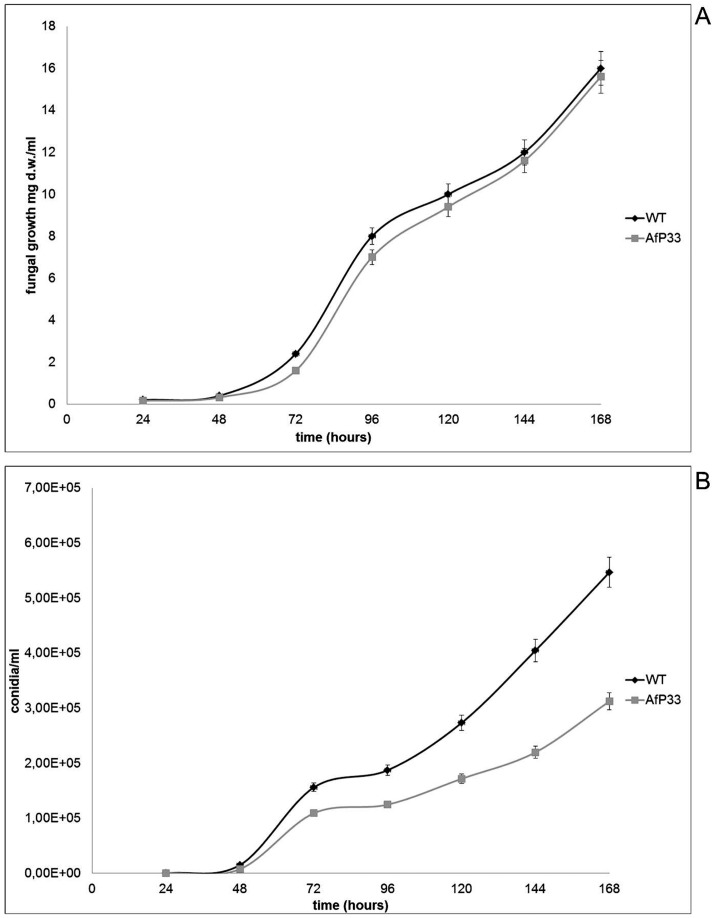
Fungal growth and conidiogenesis. A) Fungal growth (mg dry weight-d.w/mL). B) conidiogenesis (n. conidia/mL).

The insertion of the P33 gene did not apparently affect the amount and rate of growth in AfP33 strain (Figure 4A). In fact, the growth trend was in WT and AfP33 at the same level and the final amount (i.e. at 168 hpi) of biomass was similar (16.0±0.2 mg/mL in the WT vs 15.6±0.3 mg/mL in the AfP33 strain). Conversely conidiogenesis resulted significantly affected and the amount of conidia was similar up to 48 hpi (Figure 4B). After this time point the number of conidia is significantly higher in the WT strain than in the AfP33 strain. The WT strain then produced, at 168 hpi, a higher number of conidia (∼5.5×10^5^ conidia/mL) compared to the AfP33 strain (∼3×10^5^ conidia/mL)(P<0.001). The Af-DsRED strain, here used for the fluorescence microscopy characterization of peroxisomes, grew and sporulated similarly to WT (data not shown).

### *Pex11* and *foxA* expression in AfP33 and WT strains

**Figure 5:**
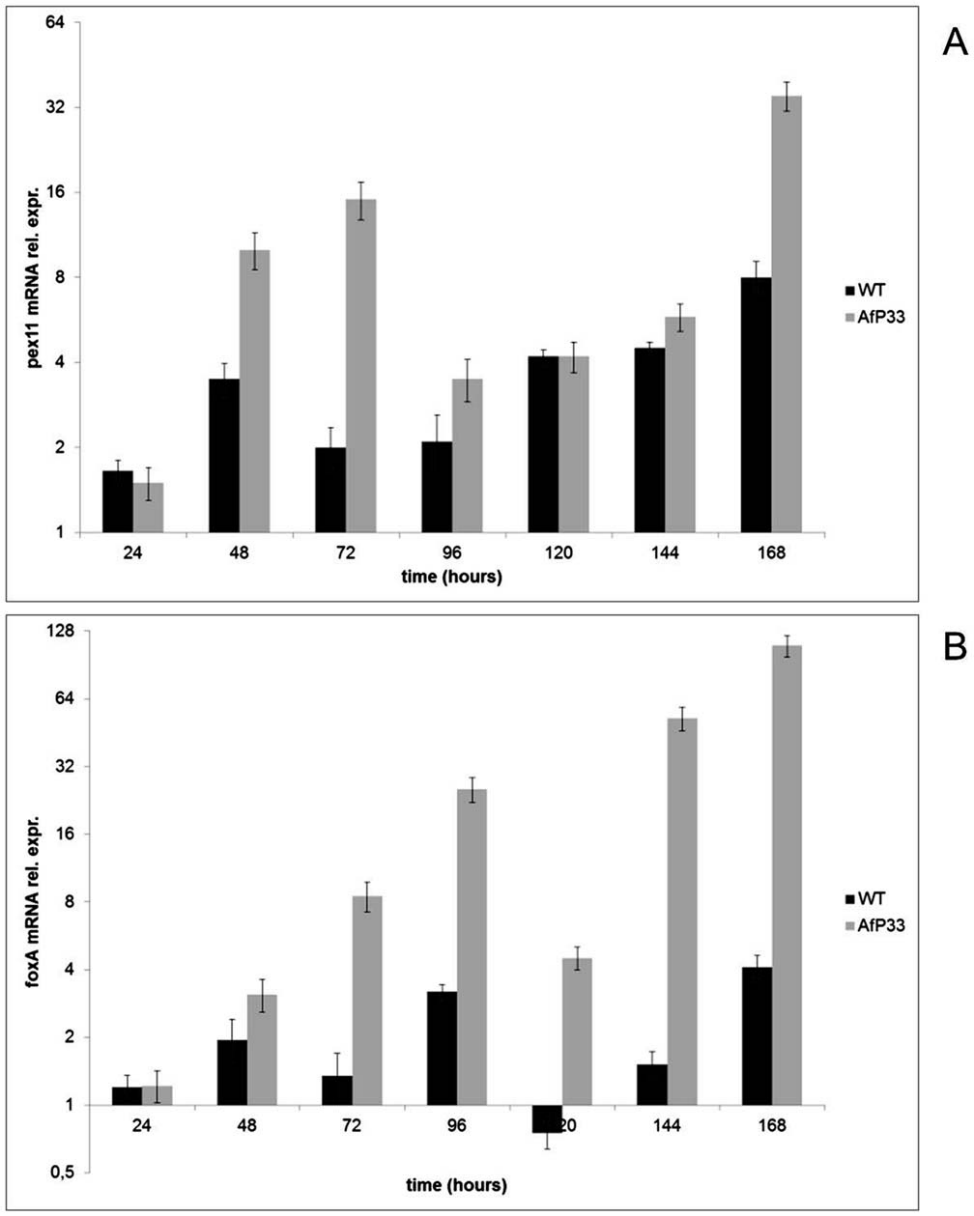
Expression of genes related to peroxisome proliferation and functionality. (A) Real Time PCR analysis of pex11 mRNA and (B) of foxA mRNA expression in AfP33 and WT strains grown in CD after different periods of incubation (24 up to 168 hpi) normalized to time 0 (inoculation).

The expression of the peroxin *pex11* mRNA (Figure 5A) and the expression of the *foxA* gene (Figure 5B), encoding for the multifunctional enzyme involved in fatty acid β-oxidation into peroxisomes [5], [21], were monitored for investigating the level of both peroxisomal proliferation and peroxisomal fatty acid β-oxidation in the AfP33 and WT strains. *Pex11*, as expected by ultrastructural analysis, was over-expressed in AfP33 strain compared to the WT strain in different time intervals (48–96 hpi and 144–168 hpi); the time points of its relative mRNA expression were correlated to P33 expression (i.e. 48–72 and 144–168 hpi; R^2^ = 0.92; P<0.01) and reached one peak at 72 hpi (Figure 5A), when an high level of expression of P33 was evident (Figures 1B–C). Furthermore, after 48 hpi, the expression of *foxA* was up-regulated in the AfP33 strain in comparison with the WT strain (Figure 4B), indicating a stimulation oftheβ-oxidation machinery related to peroxisome proliferation in this strain.

### Fatty acids metabolism

**Figure 6:**
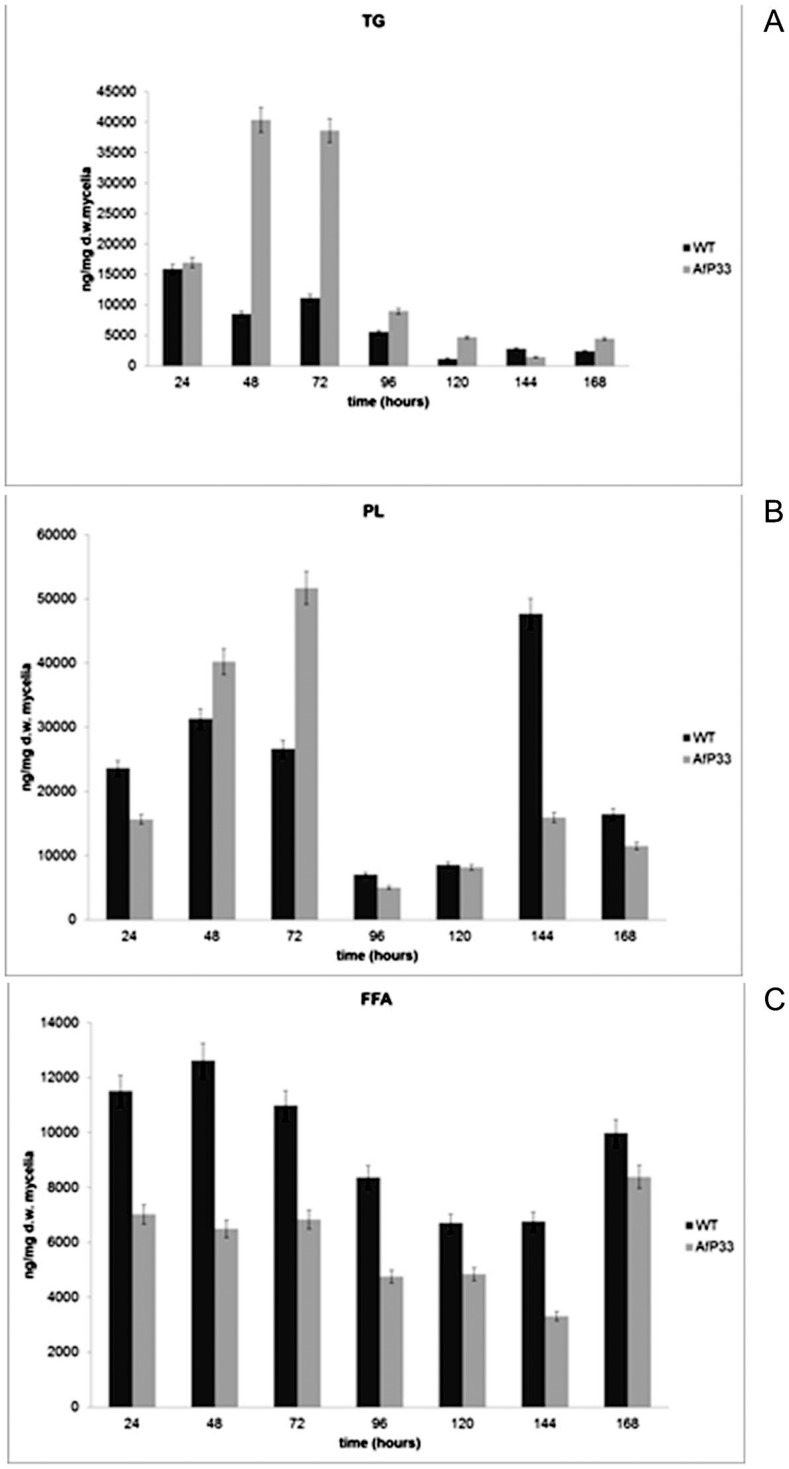
Lipid profile. (A) Total fatty acids in TG , (B) PL and (C) FFA fraction, (ng/mg dry weight-d.w.), in mycelia of WT and AfP33 strains grown in CD (25 mL) and incubated at 30°C from 24 to 168 hpi.

Since peroxisome represents a crucial site for lipid homeostasis into fungal cell, we investigated if the hyper-proliferation of the peroxisomes in AfP33 strain could affect fatty acids metabolism; thus, the amount of fatty acids in three lipid fractions, triglycerides (TG, Figure 6A), polar lipids (PL, Figure 6B) and free fatty acids (FFA, Figure 6C) was quantified. In the figures, each fraction represented the sum of the amount of hexadecanoic acid (C16:0), hexadecenoic acid (C16:1), octadecanoic acid (C18:0), octadecenoic acid (C18:1), octadecadienoic acid (C18:2) and octadecatrienoic acid (C18:3). TG (Figure 6A) amount was higher in AfP33 than in WT strain, mainly between 48 and 120 hpi. The polar lipids (PL) fraction is produced in a higher amount in AfP33 than in the WT up to 72 hpi. Thereafter, their amount in the transformant strain was lower or similar to WT (Figure 6B). The lower amount of FFA in AfP33 strain compared to the WT could be due to their faster turnover in the former, as showed by the higher rate of β-oxidation (*foxA* expression*;* Figure 5B) or by their inclusion into PL and/or TG fractions (Figures 6A–B).

### Analysis of 9- and 13-HPODE produced into *A. flavus* WT and AfP33 mycelia

**Figure 7:**
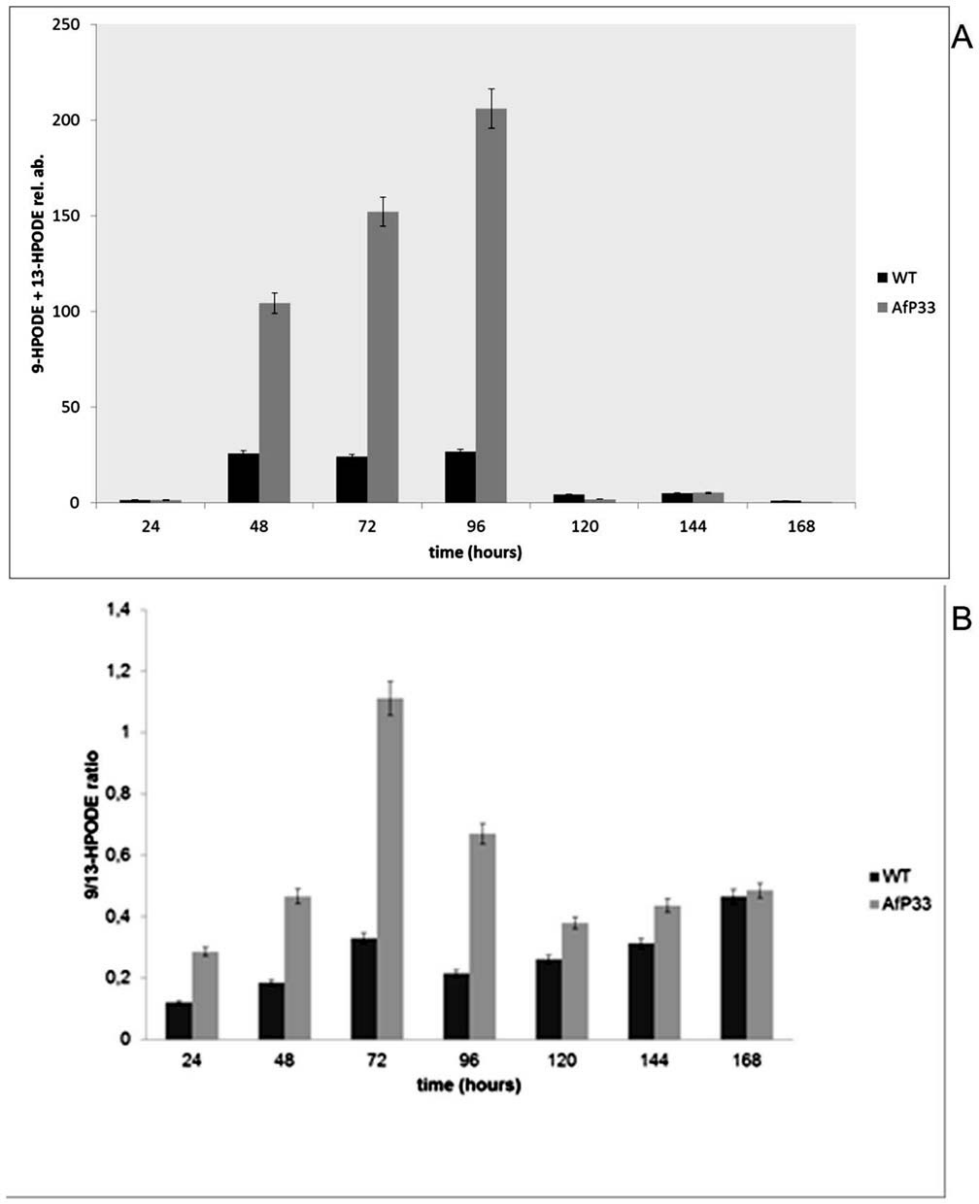
Generation of some oxylipins. (A) 9- and 13-HPODE (reported as relative abundance – rel. ab. – respect to the linoleic acid amount into the sample) and (B) 9- /13-HPODE ratio in mycelia of WT and AfP33 strains grown in CD (25 mL) and incubated at 30°C from 24 to 168 hpi.

In fungi, it was recently demonstrated that oxylipins can also be produced into peroxisomes as occurs in plants [4], [22] and we speculated that the increase of the peroxisome proliferation in AfP33 strain might lead to an enhancement of 9- and 13-HPODE synthesis. Intriguingly, in AfP33 the amount of these oxylipins was higher (P<0.01) than the WT strain (Figure 7A) at 48–96 hpi and in the transformant strain the amount of 9-HPODE was significantly higher than that of 13-HPODE in comparison to WT up to 144 hpi (P<0.01) (Figure 7B).

### Reactive oxygen species and some related scavenging activity

**Figure 8:**
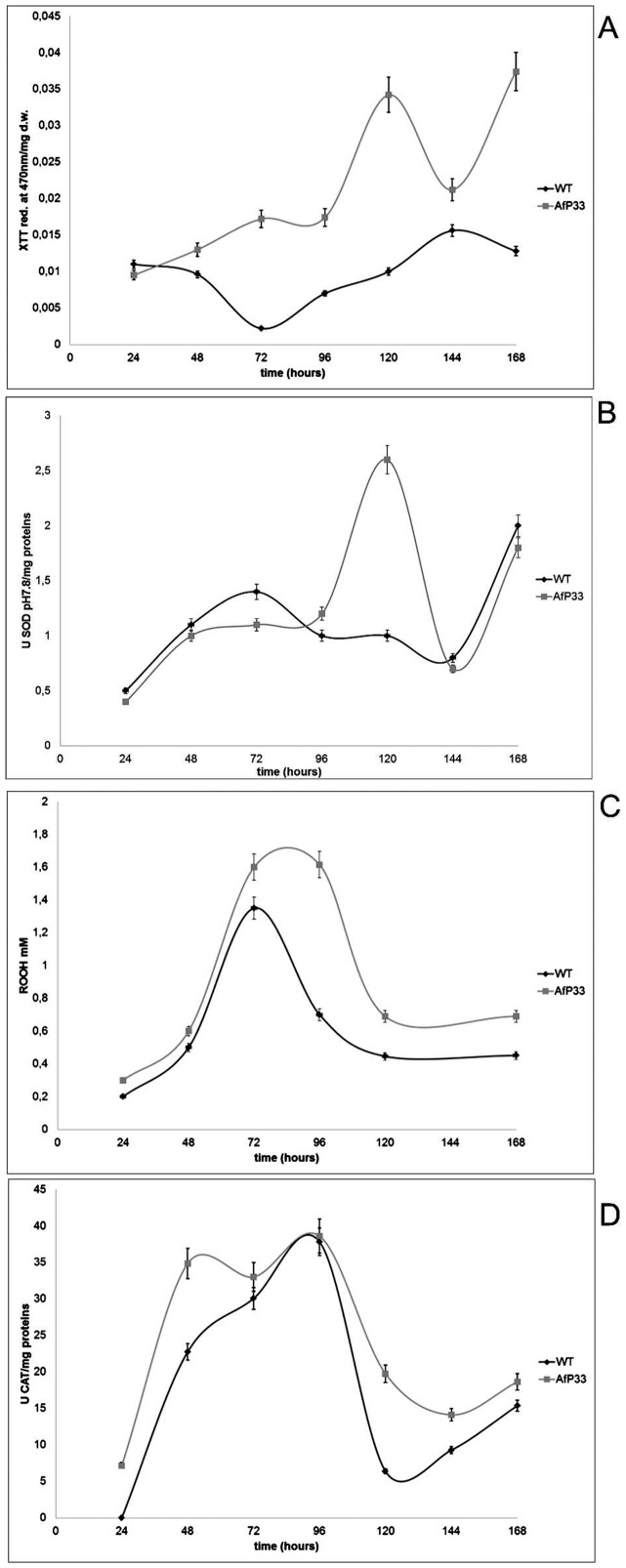
Reactive oxygen species formation and antioxidant enzyme activities. (A) Detection of superoxide anion formation (reported as absorbance of XTT formazan at 490nm/mg dry weight-d.w. mycelia), (B) SOD activity at pH 7.8 (U/mg proteins), (C) Peroxides (ROOH mM) and (D) CAT activity (U/mg protein) in mycelia of A. flavus WT and AfP33 strains grown in CD at different time intervals of incubation at 30°C.

The O_2_^•−^ level in the AfP33 strain was higher than in the WT strain (Figure 8A) during all the time intervals, starting from 48 hpi and reaching a peak at 120 hpi. Accordingly, SOD activity (Figure 8B) in AfP33 at pH 7.8 achieved its maximum at the same time (120 and 168 hpi) of the anion superoxide peak. The activity of SOD at pH 10.0 showed a similar trend but its stimulation in AfP33 occurred earlier (24–48 hpi) compared to WT (data not shown). Hydrophilic peroxides (mainly H_2_O_2_) production was increasing, in both strains, from 48 hpi and it was significantly stimulated in AfP33 from 72 hpi (P<0.01) (Figure 8C). The higher production of peroxides into the transformant strain apparently led to an up-regulation of catalase from 24 hpi. The catalase activity is stimulated at 24–72 hpi in AfP33 strain (P<0.01 for 24–48 hpi; P = 0.061 for 72 hpi) in comparison with the WT (Figure 8D).

### Aflatoxin biosynthesis

**Figure 9:**
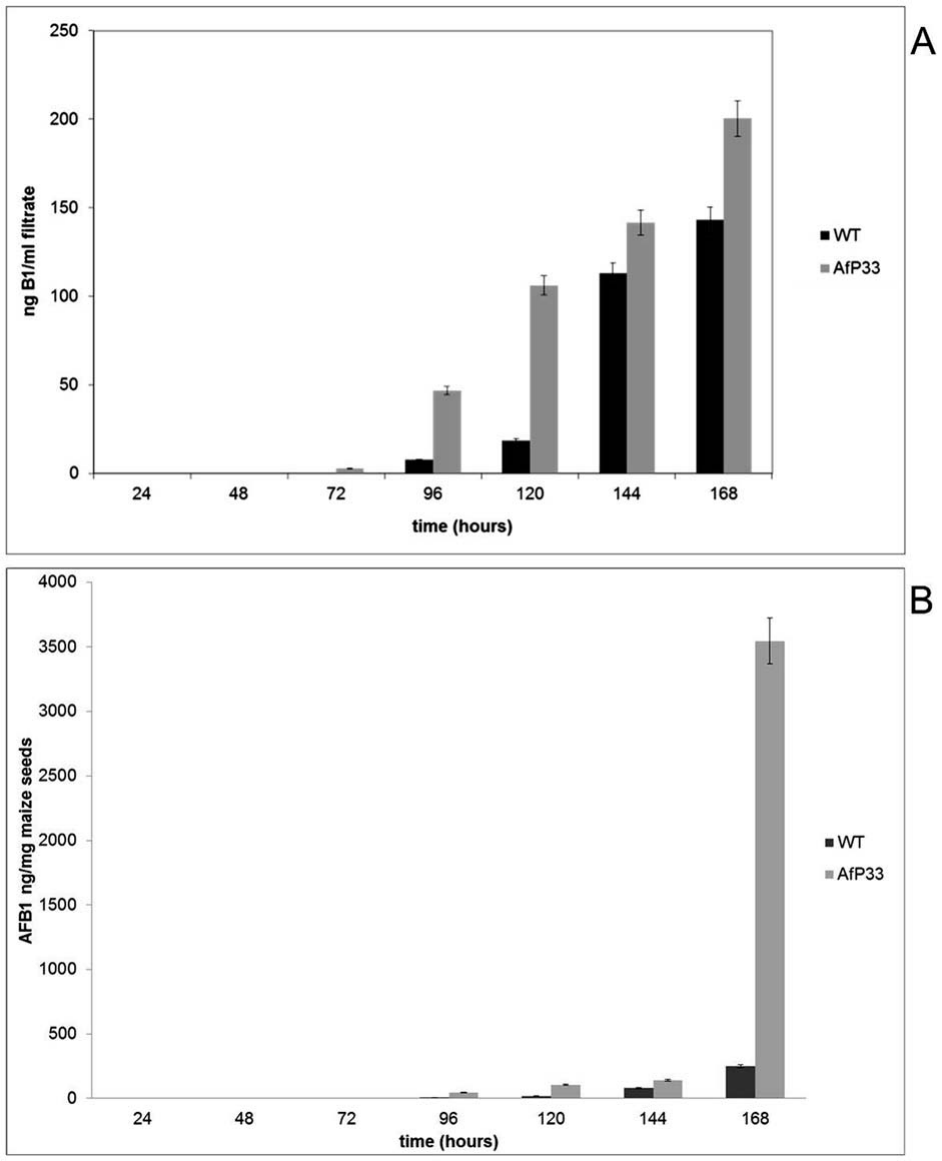
Aflatoxin synthesis in vitro and in vivo. (A) Aflatoxin (AF) production in culture filtrate (ng/mL), grown in CD after different periods of incubation. (B) Aflatoxin (AF) production in viable maize kernels (ng/mg maize seeds) in the WT and AfP33 strains at different time intervals of incubation at 30°C.

In the WT strain the aflatoxin biosynthesis started at 96 hpi, whereas in AfP33 it started one day before (1.55 ng/mL at 72 hpi – Figure 9A). AFB_1_ production *in vitro* was higher up to 7 times (∼3.5 *vs* 0.5 μg/mL) (Figure 9A) and up to 10 folds *in vivo* (Figure 9B) (∼100 *vs* 10 μg/g maize seed) compared to the WT strain.

## Discussion

Several round papers have recently gathered the knowledge of the factors that regulate AF biosynthesis in *Aspergillus* sect. Flavi [23]. Among these factors, a significant role has been fully demonstrated for lipids and their oxidised products, oxylipins [24]–[28]. Accumulating evidences suggest that mycotoxigenic pathogens and their hosts can communicate through a language in which the oxylipins are the “words” [26], [29]. In this respect, particular attention has been given to how, in *A. flavus*, lipid metabolism and AF biosynthesis are co-modulated [30]. But how do endogenous fatty acids and oxylipins affect fungal development and secondary metabolism? Probably, as suggested by Tsitsigiannis and Keller [26], these molecules act in an autochrine/parachrine fashion modulating these events through GPCR-FadA-PkaA signalling. Previous studies indicate that long-chain saturated free fatty acids (FFA) from lauric (C12:0) to behenic (C22:0) support *A. flavus* growth without affecting AF biosynthesis, whereas caprylic (C8:0), nonanoic (C9:0) and undecanoic acid (C11:0) are toxic for the fungus. Unsaturated fatty acids (UFA) also affect *A. flavus* having a greater inhibitory effect on the growth and, conversely, a higher stimulating effect on AF synthesis. In fact, the product of UFA/PUFA oxidation, i.e. the oxylipins, can be responsible for mycotoxins and in particular for AF modulation [13]; [25]; for instance, 9-hydroperoxyoctadecenoic acid (HPODE) stimulates, whereas 13-HPODE inhibits, the expression of some genes, i.e. *aflR* and *aflK*, related to AF biosynthesis [27], [30]. In fungi, peroxisomes play a central role in fatty acids metabolism (β-oxidation) and they begin to proliferate and enhance the fatty acid β-oxidation machinery during the interaction with the host or under nutrient depletion [8]. Over recent year it is emerging the role of peroxisomes as regulator not only of the lipid metabolism, but also of the cell redox balance and of the synthesis of secondary metabolites in fungi [31]. Accordingly, Maggio-Hall et al. [5] showed that peroxisomes play a role in AF biosynthesis by *A. flavus.* In relation to this, norsolorinic acid, the aflatoxin precursor, was reported to accumulate into these organelles, even if it is unclear how peroxisome proliferation stimulate AF synthesis. Other examples of the relationship between peroxisome proliferation and the synthesis of secondary metabolism has been further reported in *Penicillium chrysogenum*. In this fungus an increase of the peroxisomes number, obtained by the genetic manipulation of the peroxisome proliferator Pex11p, enhanced the production of penicillin [4]. More recently, a comparison of various strains of *P. chrysogenum*, selected during studies of lineage improvement, have revealed that high penicillin producer strains contained increased number of peroxisomes in comparison with the low producing ones [6]. The Pex11p factor, a member of the large peroxin family, is the main regulator of peroxisome proliferation in fungi [21]; this peroxisome-associated protein shows extensive amino acid sequence similarity to the ligand-binding domain (LBD) of the peroxisome proliferators activated receptors (PPAR). PPAR, in metazoans, are the key regulators of FA metabolism activated by FA and oxidised FA (such as 9 and 13-HODE) [32], [33].

Thus, in our paper, we want, firstly, to demonstrate that the up-regulation of AF synthesis occurs via proliferation of peroxisome. In relation to this, we have amended *A. flavus* cultures with a PPARα agonist (BEZAfibrate) and monitored *pex11* and *foxA* gene expression and AF production.

As suspected BEZA enhances *pex11* and *foxA* gene expression and also AF synthesis in *A. flavus*. This result is a clear hint that a correlation between peroxisome proliferation and AF production takes place in *A. flavus.*

Anyway, the question is, why the proliferation of peroxisomes enhances AF synthesis? It is also known that BEZA triggers the β-oxidation of fatty acids [34] and that acetate is needed for AF synthesis [5], [35] and, in relation to this, the enhancement of β-oxidation can fulfil this necessity; nevertheless, several papers have clearly shown that oxidative stress is a prerequisite for AF synthesis [12]–[14]. Being peroxisomes also a site for ROS and oxylipins generation [4], [36], [37], we sought the existence of a correlation among peroxisomal proliferation, the alteration of the cell redox balance, the formation of oxylipins and the biosynthesis of AF in *A. flavus*. In fact, pro-oxidants such as cumene hydroperoxide and hydrogen peroxide trigger AF synthesis in *A. flavus* too (Figure S2).

By transforming *A. flavus* with the viral gene *P33* – whose product is a peroxisome proliferator [18] – and by controlling its transcription with the promoter of the Cu, Zn-superoxide dismutase gene of *A. flavus*, we directly link, in the AfP33 transformant strain, peroxisomes proliferation with the up-regulation of the Cu, Zn-*sod* subsequent to the increase of ROS inside the cell [13]. The effect of P33 has never been studied in filamentous fungi, however in *Saccharomyces cerevisiae* it promotes the peroxisomes number and the proliferation of their membranes [17], [18]. The same effects are reported when the human cells are treated with bezafibrate which activate PPAR [38] that, in turn, controls hepatic peroxisome proliferation [39]. This drug, *via* PPAR activation, also stimulates β-oxidation of fatty acids in peroxisomes favouring an hypolipidemic effect.

We supposed that P33, by stimulating the number and the proliferation of fungal peroxisomes, could also affects, as bezafibrate in humans, the fatty acids oxidation., In fact, we demonstrated that P33 enhances the expression of *fox A* which is correlated with the β-oxidation in fungal peroxisomes and, for this, is considered as peroxisomal beta-oxidation marker [5].

Our results indicate that the AfP33 strain, which presents peroxisome proliferation, shows an higher turnover of FFA which can be targeted to their higher utilization in the PL formation, to the peroxisomal β-oxidation (as suggested by the increase of the multifunctional β-oxidation *foxA* gene expression) and to the formation of oxylipins. Moreover, an higher level of oxidants (e.g. superoxide anions and peroxides), a significant alteration of the antioxidant profile (e.g. SOD and CAT) was evidenced in AfP33 compared to the WT strain. It can be suggested that the amount of ROS, increased consequently to peroxisome proliferation in the AfP33 strain, overcomes the threshold of ROS “supported” by this strain and a marked AF increase occurs. This hypothesis would be in agreement with the point of view elaborated by other authors (Linz et al., personal communication) [13] that consider AF as “defence” molecules synthesised by A. flavus to cope with the excess of unscavenged toxic ROS in the late phase of growth. Growing evidences, in other systems, support the view that peroxisomes not only control the intra-peroxisomal homeostasis of ROS but can also contribute to the maintenance of extra-peroxisomal ROS levels within the entire cell [37]. Thus, peroxisomes in mycotoxigenic fungi can play an important role to establish oxidative intracellular conditions and to generate the oxylipin signals which are needed for the progression of toxin synthesis [26], [40].

By this report we define the role played by peroxisomes, as significant modulators of reactive oxygen species, oxylipins and, in turn, of aflatoxin biosynthesis in A. flavus.

## Materials and Methods

### Fungal strains and culture conditions

*Aspergillus flavus* NRRL 3357 (WT), aflatoxin B_1_ producer and an uracil auxotroph mutant (3357-5) [41] were used in these studies. An AfproSODP33GFPDsRED (from here abbreviated as Afp33 – see Table 2) transformant was generated from 3357-5 as described below. The fungal strains were maintained on Czapek Dox Agar (CDA) (Difco), amended with ZnSO_4_ (5 mg/L) and NaMoO_4_ (1mg/L), for 7 days at 30°C. Fifty ml of Czapek Dox Broth (CDB) (Difco, USA) in 100 mL Erlenmeyer flasks were inoculated with the WT or AfP33, using 0.1 mL of conidial suspension (∼10^6^ conidia) for each flask; the cultures were then incubated at 30°C for different time periods (24, 48, 72, 96, 120, 144, 168 hpi).

**Table 2:** *A. flavus* strains used in this study.

Strains	Genotype	Plasmids used	Ref.
3357	Wild type		ATCC collection
3357.5	*pyrG^-^*		Zhu-Mei et al., 2007
AfproSODP33	Af*proSOD*::*P33*::*pyrG*	pUC19::0,8 Kb Cu,Zn SOD promoter region; pYESP33; pUC19::proSOD::P33; pBSK-*pyrG*-alcA	This study
AfP33	Af*proSOD*::*P33*::*pyrGDsRED::hygromycin*	PZ4DsREDSKL; pSILENT-1 pSDsRed	This study
Af-DsRED	*DsRED::hygromycin*	PZ4DsREDSKL; pSILENT-1 pSDsRed	This study

### Fungal growth, conidiogenesis and aflatoxin production in culture media

At each point in time, fungal growth was determined by weighting the mycelium after filtration (Millipore filters, 0.45 μm) and drying it for 48 h at 80°C (d.w.). To determine the amount of ROS and oxylipins and to perform molecular analyses, the filtered mycelia were lyophilized and weighed. Conidiation was determined at each time interval by completely washing the mycelia with a solution of triton 0,01% w/v, picking up 0.5 ml of this solution and calculating conidia's number using a hemacytometer. In other experiments BEZAfibrate (BEZA; Sigma-Aldrich, USA), a peroxisome proliferator in mammals *via* PPAR, was added at concentration of 1mM to the *A. flavus* cultures and *pex11* expression and AF production was monitored. Aflatoxin production was analysed from cultures of the WT and AfP33 strains grown both in CDB and onto viable maize seeds following extraction with chloroform:methanol (2∶1 v/v). The extracts were collected; the volume was reduced under a stream of nitrogen and the quantitative analyses were carried out by HPLC, as previously reported [13].

### Lipid analysis

WT and AfP33 lyophilised mycelia (10 mg) collected from CDB cultures at different incubation times (from 24 to 168 hours) were homogenized in liquid nitrogen to avoid the accidental formation of peroxides. To analyse the lipid content, the mycelia were extracted three times with 1mL CHCl_3_/CH_3_OH (2∶1, v/v) in the presence of 100 µg of butylated hydroxytoluene (BHT) as an antioxidant. Nonadecanoic acid (C19:0) was added as internal standard before extraction. After filtration and evaporation under vacuum, the extracted lipids were fractionated by thin layer chromatography (TLC) and the polar lipid (PL), the triglyceride (TG) and free fatty acid (FFA) fractions were recovered as previously reported [42] with slight modifications. The extracted lipids were trans-esterified by boron-trifluoride (BF_3_ 10% in CH_3_OH; Merck, Germany). The resulting fatty acid methyl-esters were analysed by gas chromatography (GC) on a capillary column FFA-P 50 m x 0.32 mm x 0.52 µm [43].

### Quantification of total peroxides in mycelia (FOX-1 assay)

The hydroperoxides produced by WT and AfP33 strains grown in CDB medium at different incubation times (from 24 to 168 hours) was analysed by spectrophotometric assay by monitoring the oxidation of xylenol orange (FOX-1) at 560 nm. The sensitivity of the method was increased by the use of triphenyl-phosphine and stabilizing the reagent at pH 1.7–1.8, as reported by Reverberi et al. [13].

### Analysis of 9- and 13- HPODE in mycelia

The WT and AfP33 collected from CDB cultures at different incubation times were homogenized in liquid nitrogen to avoid the accidental formation of peroxides. Hydroperoxyoctadecadienoic acids (9- and 13-HPODE) present in the mycelia were analysed following extraction with chloroform:methanol (2∶1 v/v), three times, in the presence of 100 μg of butylated hydroxytoluene (BHT) as antioxidant. Peroxides in the extracts were reduced with NaBH_4_ to obtain hydroxyoctadecadienoic acids (9- and 13-HODE). Neither 9- nor 13-HODE were detected in the extracts before reduction. The regioisomers 9-HODE and 13-HODE were analysed by HPLC-APCI-MS and their amount has been expressed as relative to linoleic acid, their natural substrate, as reported previously [13]. 9-HODE and 13-HODE were identified by comparison with authentic specimens (purchased from Cayman, USA).

### Quantification of anion superoxide in mycelia

The WT and AfP33 lyophilised mycelia (10 mg) collected from CDB cultures at different incubation times (from 24 to 168 hours) were homogenized as reported above. O_2_^−•^ accumulation was measured at each point in time. O_2_^−•^ was detected by measuring the reduction of 2,3-bis(2-metoxy-4-nitro-5-sulphophenyl)-5-[(phenylamino) carbonyl]-2H-tetrazolium hydroxide (XTT) to formazan, according to Reverberi et al. [13]. Absorbance at 490 nm was measured in a microplate reader (μQuant Bio-Tek instruments). XTT levels were expressed as mean absorbance values/mg ± S.E.M.

### Activities of superoxide dismutase and peroxide reducing enzymes

The activities of SOD pH 7.8 and pH 10.0 (EC 1.15.1.1), catalase (CAT) (EC 1.11.1.6) enzymes were analysed as previously described [13] in WT and AfP33 strains.

### Ultrastructural (TEM), fluorescence microscopy characterisation

Fungal strains were washed three times in PBS and fixed with 2% glutaraldehyde in PBS for 2 hours at 25°C. Samples were post fixed in 1% osmium tetroxide in veronal acetate buffer (pH 7.4) for 12 hour at 4°C, stained with uranyl acetate (5 mg/ml) in veronal acetate buffer, pH 6.0, for 1 hour at 25°C and then were dehydrated in ethanol and embedded in Epon 812. Thin sections were examined unstained or post-stained with uranyl acetate and lead hydroxide. Fungal strains, plated on coverslips previously coated with 2% gelatin onto 24-well plates, were fixed in 4% parafomaldehyde in PBS for 30 min at 25°C and permeabilized with 0.1% Triton X-100 in PBS for 5 min. Nuclei were visualized using DAPI (Sigma Chemicals Co., St. Louis, MD). Fluorescence signals were analyzed either by a Zeiss Fluorescence Microscopy (Zeiss, Axioskop 2 Plus, Oberkochen, Germany) recording stained images using a CCD camera (Zeiss, Oberkochen, Germany) and IAS2000/H1 software (Delta Sistemi, Italy).

### Plasmids and transformation

A survey of plasmids and strains used in this paper are shown in Table 2. DNA extracted from *A. flavus* NRRL 3357 was amplified in a thermal mastercycler gradient (Eppendorf) following amplification steps (94°C×2 min; 94°C x 30 s, 65°C×45 s, 72°C×1 min x 35 times; 72°C×8 min) using the primers Afsodp (forward 5′-CTGCAGGTGTCGATCTGGAGCTATCG-3′; reverse 5′-CTGCAGGCTAGCTCAAGTGAAGGGAAGATAGATTGA-3′) designed in the 1.0 Kb upstream the sequence AFLA099000 present in the EQ963484.1 region of *A. flavus* (www.aspergillusflavus.org). Amplification under high stringency conditions produced a unique band for *SOD* promoter fragment (0.8 Kb, gene bank accession number DQ104418). The forward primer presented *Pst*I sequence (*forAfsodpPst*I 5′-CTGCAGGTGTCGATCTGGAGCTATCG-3′) while the reverse primer presented *Pst*I and *Nhe*I sequences (*revAfsodpPst*I*Nhe*I 5′-CTGCAGGCT GCTCA AGTGAAGGGAAGATAGATTGA-3′). This feature allowed the insertion of the *SOD* promoter in pUC19 in the *Pst*I site, and allowed subsequently the insertion of *p33* in the *Nhe*I site. The *SOD* promoter was cloned into pGEM-T easy vector (Promega, USA), then restricted with *Pst*I and inserted in the plasmid pUC19 previously restricted with *Pst*I. The plasmid pYESP33 containing the viral gene *p33* was kindly provided by Dr. L. Rubino (Università di Bari, Italy). A PCR was performed for *p33* with two primers with restriction sequences at the end: both the primers forward and reverse had *Nhe*I sequence (*For2p33* 5′-GCTAGCCTTGGAAATCCTCCAGGACA-3′ and *Rev2NheIp33*5′-GCTAGCCGCAAATTAAAGCCTTCGAG-3′). The resulting *Nhe*I – restricted 2.1 Kb fragment corresponded to the entire fragment *p33* (in pYES2). This included the terminator sequence as well. This fragment (*p33*) was ligated into *Nhe*I-restricted pUC19+*SOD* promoter for creating the plasmid subsequently used for transforming AF3357-5 together with the plasmid pBSK-*pyrG* (which complements the uracil autotrophy) and creating AfP33 strains. The ∼3.0Kb fragment AFLA099000pro-P33 contains an *EcoR*I site at the 3′-end.

In order to highlight peroxisome proliferation, *A. flavus* WT and AFP33 strains were transformed with a plasmid containing the gene encoding the red fluorescent protein (DsRED) fused with the peroxisome targeting signal -SKL (PTS1). The DsRED-SKL fragment (0.7 Kb) was obtained by amplifying the plasmid pZ4-DsRED-SKL (6.55 Kb), kindly provided by Dr. J.A.K.W Kiel (University of Groningen, The Netherlands), with primers forward (*forHin*dIII) *5* ′-AAGCTTGGATCCATGGCCTCC-*3*′; reverse (*revBgl*II)*5*′-AGATCTTTACAGCTTCGACTTG-*3*′. The PCR fragment was inserted into pGEMT-easy, restricted with *Hin*dIII and *Bgl*II and then inserted into pSILENT-1 vector (6.7 Kb) alongside the PtrpC and TtrpC, promoter and terminator region of the *hph* (hygromycin B phosphotransferase) cassette for obtaining the pSDsRED plasmid (7.4 Kb) containing also the *hph* resistance cassette.

### Selection of transformants

The selection of AfP33 transformant strains was conducted at 30°C on Czapek Dox agar (CDA) containing 30 mM arginine as the sole nitrogen source; putative transformants were selected, transferred to fresh selective medium, and allowed to sporulate. To obtain homokaryons, single spores were isolated from each selected heterokaryotic transformant and transferred to fresh selective medium. This monoconidial transfer was conducted three times. Finally, 20 monoconidial progenies were selected and further sub-cultured to determine the occurrence of abortive transformants. The stability of these transformants was also tested by two additional single-spore transfers on non-selective medium and then again on selective medium, and by several mycelial transfers on selective plates. The protoplasts obtained from conidia of stable AfP33 tranformants were transformed with a 4.7 Kb *Stu*I*-Xba*I fragment excised from pSDsRED, which also carries the hygromycin B resistance selectable marker. The stability of these double transformants AfP33 was tested by several single-spore transfers on selective media (Hyg B 500 ppm), as described above for AfP33 selection.

### Southern and western blot hybridisation

For Southern blot analysis, 10 μg of genomic DNA from *A. flavus* NRRL 3357 and AfP33DsRED was completely restricted with *Eco*RI (10 U) at 37°C for 4 h in the manufacturer's buffer at the recommended concentrations (Fermentas, Germany). *EcoR*I-digested DNA fragments were separated by electrophoresis for 3 h and 30 min at 40V on 0.8% w/v agarose gel in TAE buffer. DIG-labelled *Hin*dIII cut lambda (λ) (Roche, Swiss) was used as MW standard. Fluorescent DNA probes were prepared according to the PCR DIG-labelling mix method (Roche, Swiss). The membranes were pre-hybridized according to the instructions of the manufacturer of the DIG- detection kit, at 64°C in DIG-easy buffer (Roche, Swiss); they were then hybridized for 12-16 h in the same buffer containing 250 ng of freshly denatured digoxigenin *P33* probe at 65°C.

Total protein samples (16 μg) were electrophoresed on 12% polyacrylamide-SDS gels and subsequently blotted in a blot apparatus onto nitrocellulose in 25 mM Tris, 192 mM Glycine, pH 8.3. Blots were blocked by immersing the membrane in 5% dried milk in a buffer containing 1% Tween 20, NaCl 150 mM, Tris-HCl pH 8.0 20 mM for 1 h at room temperature, subsequently incubated with P33 antiserum o/n at 4°C, and finally incubated with rabbit anti-goat peroxidase conjugate for 1 h. Blots were developed using the ECL detection system as described by the manufacturer (Amersham, USA).

### RT-PCR analyses

Total RNA from 100 mg of freeze-dried mycelia was extracted using the Tri-Reagent protocol (Sigma-Aldrich, USA) and was quantified by spectrophotometry, determining the optical density at 260 nm. RNA was treated with RNAse-free DNAse I and then re-suspended in 20 μL of DEPC-treated water. RNA was extracted at different points in time (from 24 to 168 hpi; 3 tubes for each point in time) from *A. flavus* WT and AfP33DsRED CDB cultures and was used to develop *p33*, *pex11* (in presence and in absence of BEZA 1 mM) and *foxA* (primer sequences in Table 3) SYBR green RT-PCR assay, as previously reported [44]. Gene expression in the WT strain and the AfP33 transformants were also measured by comparing mRNA levels in the different time intervals with their own basal expressions at the baseline, i.e., after conidia germination (time 0). *A. flavus* β-tubulin RNA was used as the housekeeping gene to normalize the differences in total RNA target input and quality and in RT efficiency, using specific primers as Afβtub (Table 3).

**Table 3:** Primers used in this study.

Oligo Name	Sequence (5′-3′)
Afβtub_for	GGAAGTCAGAAGCAGCCATC
Afβtub_rev	GTGACCACCTGTCTCCGTTT
p33_for	TGTGCTGCTAAGTGGGAATG
p33_rev	TCCTCCACTCCATCCTGGTA
pex11_for	TCGACCCCTACAATGCAGTC
pex11_rev	CTGCAGCGCTATTGCTAGTG
foxA_for	AGACCGCCATTGACACCTAC
foxA_rev	CACGGGAGAATCCGAGAATA
AfSOD1_for	CATGTCCACCAGTTCGGTGA
AfSOD1_rev	TGTTCACTACGGCCAAGGTC

### Phenotype Array (Biolog^tm^)

The Biolog Phenotype MicroArray (PM) technique (Biolog Inc.,Hayward, CA) was used to investigate the effects of mutation on carbon assimilation patterns. To prepare the inoculum, the strains of the WT and AfP33 were pre-cultivated on 2% MEA plates for 7 days. Inocula were prepared by rolling a sterile, wetted cotton swab over sporulating areas of the plates. The conidia were then suspended in sterile Biolog FF inoculating fluid (0.25% Phytagel, 0.03% Tween 40), gently mixed, and adjusted to a transmission of 75% at 590 nm. One hundred microliters of this conidial suspension was then dispensed into each of the wells of the PM plates, and the plates were incubated with constant darkness at 30°C. The optical density at 750 nm (OD750) (mycelial growth) and OD490 (mitochondrial activity) were measured after 24, 48, 72, 96,168, 192 and 240 h using a microplate reader (Biolog Inc., Hayward, CA). The OD750 at 48 h was chosen as a reference time point for the growth assays, since it allowed a comparison of biomass formation on all carbon sources when the majority of growth curves were in the linear phase. By using the ERGO light software (www.ergo-light.com) all the compounds present in the bio-log plates were assigned to specific metabolic pathways. The pathway utilisation were compared in the two strains by comparing the mean values (± S.E.) of the substrates present by student T test.

### Statistics

All the experiments were carried out in three replicates of two biological replica. The values presented in figures and tables are the mean ± S.E.M. of 6 different results. The mean values were compared by using the Mann-Whitney test; *p* values above 0.05 were considered not significant. Analysis of variance (ANOVA) was applied in the comparison of the treatments, and significance of differences were tested at 95% confidence by Fisher's LSD test which is a least significant difference (LSD) method consisting in a two-step testing procedure for pair wise comparisons of several treatment groups. Calculations were performed using XLSTAT Addinsoft software [45].

## Supporting Information

Figure S1 ***P33, foxA,*** **pex11 mRNA expression and AFB1 biosynthesis of AfP33 transformants**. A) Real Time PCR analysis of *P33,* (B) *foxA* mRNA and (C) of *pex11* mRNA expression in AfP33 strains (#3, #4 and #1 as indicated in Figure 1) grown in CD for 7 days after inoculation. (D) Aflatoxin (AF) production in culture filtrate (ng/mL) of AfP33 strains (#3, #4 and #1 as indicated in Figure 1) grown in CD after 7 days after inoculation. The results are expressed as folds compared to the AfP33 strain#2 used for the rest of the experiments.
(TIF)

Figure S2 **Aflatoxin synthesis and peroxides production by** ***A. flavus*** **WT strain in liquid cultures amended and not amended with oxidants.** (A) Aflatoxin (AF) biosynthesis (ng/mL) and (B) peroxides (ROOH mM) production in *A. flavus* WT strain grown in CD not amended (CD) or amended with cumene hydroperoxide (1 mM) or hydrogen peroxide (10 mM) after different periods of incubation (3–7 dpi) at 30°C.
(TIF)

Table S1 **Analysis of the regulatory elements present in the 2.0 Kb 5' Flanking sequence (upstream) of AFLA099000, retrieved from** http://fungi.ensembl.org/Aspergillus_flavus/Info/Index **, performed with the genomic tools present in the aspergillusflavus.org website and through the NSITE tool present in the softberry.com website.**
(DOCX)

Table S2 **Analysis of the effect of P33 on different aspects of the** ***A. flavus*** **metabolism by the mean of a phenotypic microarray based approach.** All the results were analysed by XLSTAT 2007.6 -discriminant Analysis (DA) – and Principal Component Analysis (PCA). By using the ERGO light software (www.ergo-light.com) all the compounds present in the bio-log plates were assigned to specific metabolic pathways. The pathway utilisation were compared in the two strains by comparing the mean values (± S.E.) of the substrates present by student T test.
(XLSM)
