# Supplementary figures and images for: How Peroxisomes Affect Aflatoxin Biosynthesis in Aspergillus Flavus
Source: PLoS One. 2012 Oct 19;7(10):e48097. doi: 10.1371/journal.pone.0048097 (PMC3477134; doi:10.1371/journal.pone.0048097)

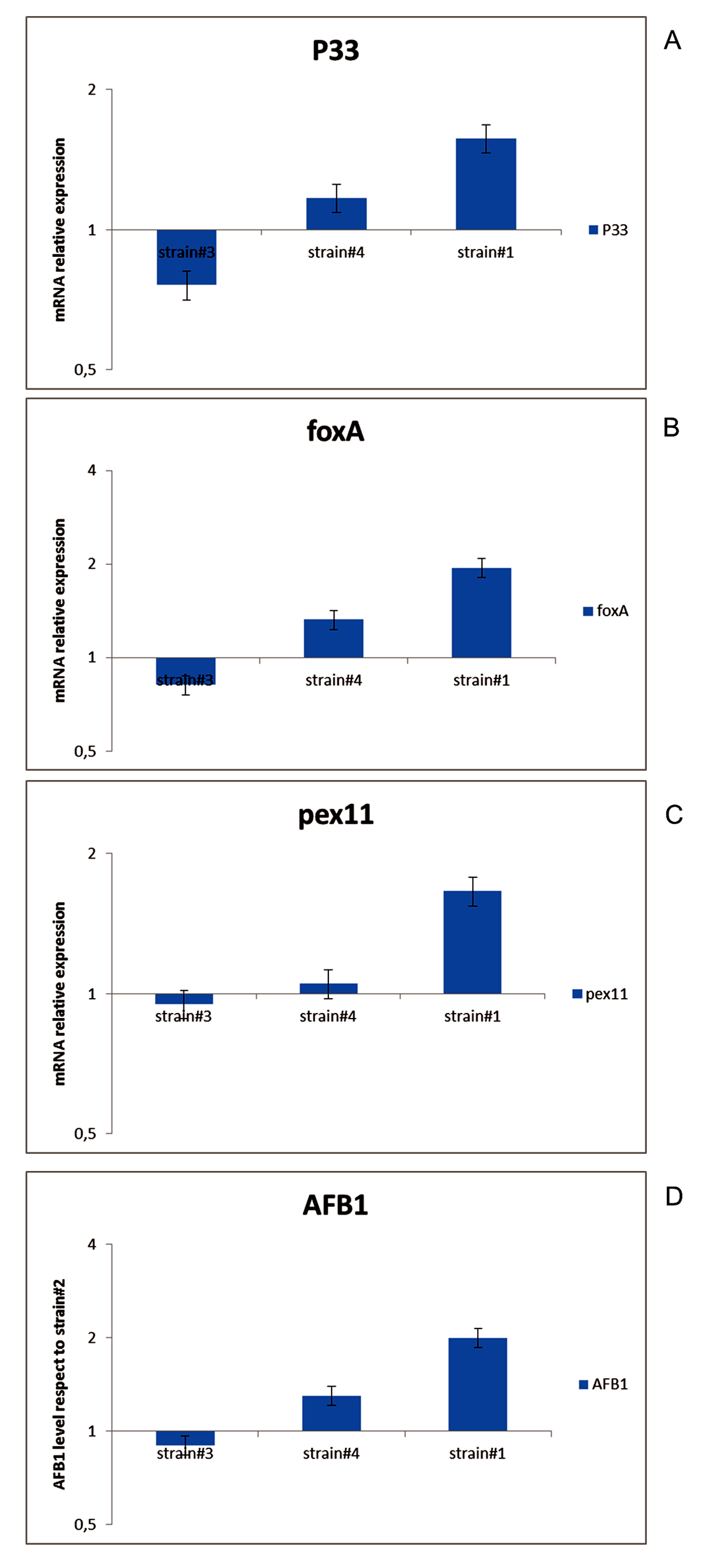

Supplement: Figure S1 — P33, foxA, pex11 mRNA expression and AFB1 biosynthesis of AfP33 transformants. A) Real Time PCR analysis of P33, (B) foxA mRNA and (C) of pex11 mRNA expression in AfP33 strains (#3, #4 and #1 as indicated in Figure 1) grown in CD for 7 days after inoculation. (D) Aflatoxin (AF) production in culture filtrate (ng/mL) of AfP33 strains (#3, #4 and #1 as indicated in Figure 1) grown in CD after 7 days after inoculation. The results are expressed as folds compared to the AfP33 strain#2 used for the rest of the experiments. (TIF) [file pone.0048097.s001.tif]

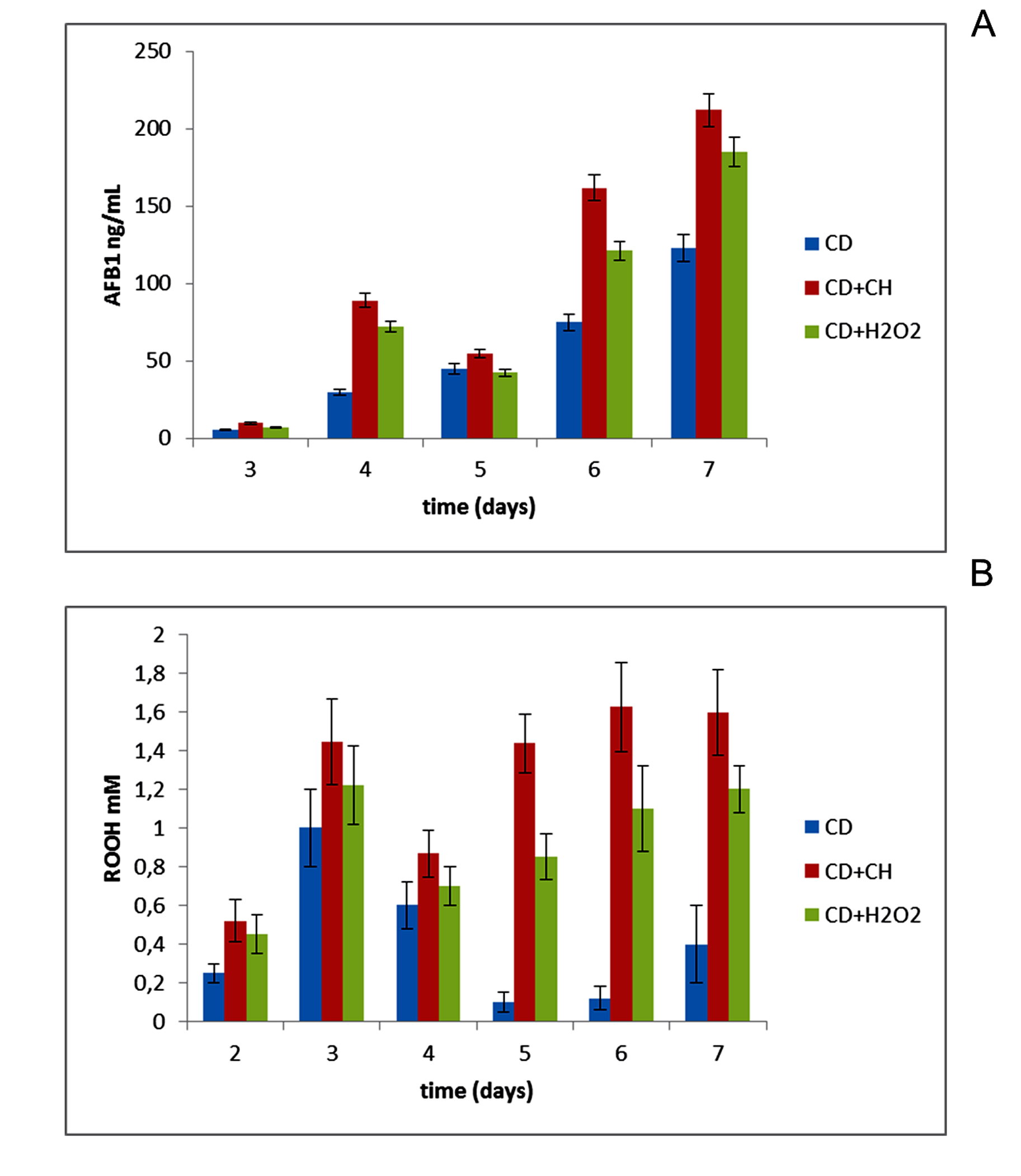

Supplement: Figure S2 — Aflatoxin synthesis and peroxides production by A. flavus WT strain in liquid cultures amended and not amended with oxidants. (A) Aflatoxin (AF) biosynthesis (ng/mL) and (B) peroxides (ROOH mM) production in A. flavus WT strain grown in CD not amended (CD) or amended with cumene hydroperoxide (1 mM) or hydrogen peroxide (10 mM) after different periods of incubation (3–7 dpi) at 30°C. (TIF) [file pone.0048097.s002.tif]
