# Supplementary material for: How Peroxisomes Affect Aflatoxin Biosynthesis in Aspergillus Flavus
Source: PLoS One. 2012 Oct 19;7(10):e48097. doi: 10.1371/journal.pone.0048097 (PMC3477134; doi:10.1371/journal.pone.0048097)
Supplement: Table S1 — Analysis of the regulatory elements present in the 2.0 Kb 5' Flanking sequence (upstream) of AFLA099000, retrieved from http://fungi.ensembl.org/Aspergillus_flavus/Info/Index , performed with the genomic tools present in the aspergillusflavus.org website and through the NSITE tool present in the softberry.com website. (DOCX) [file pone.0048097.s003.docx]

**Table S1.** Analysis of the regulatory elements present in the 2.0 Kb 5' Flanking sequence (upstream) of AFLA099000, retrieved from http://fungi.ensembl.org/Aspergillus_flavus/Info/Index, performed with the genomic tools present in the aspergillusflavus.org website and through the NSITE tool present in the softberry.com website.

>supercontig:JCVI-afl1-v2.0:EQ963484:473407:476791:-1

CACGTACGTCATCCTTGAGTTTTCTGCATTTTATTTTCCATATTGGAGTACTCGTCTAAC

AGTATAAATAGGGCAGCAACGTCGACGAGAAGGTCGACGCCACCCTTGACCGTCTCCTGG

TTGAGTTCGGCAAGAAGATCCTCGAGATCATCCCCGGAAAGGTCTCCACTGAGGTTGATG

CTCGCCTCTCTTTCGACACCCAGGCTTCCATCGACAAGGCCCTCCACATCATCAAGGTAT

GATATAATCAGATTAATATAGGTCTGGCGAGCGTGCAGTTAACATTACATATAGCTCTAC

GAGGAGAACGGTATCTCTAAGGACCGTGTCCTGATTAAGATCGCCTCCACCTGGGAGGGT

ATCAAGGCCGCTCAGGTCCTCCAGCGTGACCACGGCATCAACTGCAACCTGACCCTCATG

TTCTCCACCGTCCAGGCCATCGCTGCTGCCGAGGCCGGTGCCTACCTCATCTCTCCTTTC

GTTGGCCGTATCCTTGACTGGTACAAGGCTGCCCACAAGCGTGACTACACTGCCCAGGAG

GACCCCGGTGTTAAGTCCGTCCAGAACATCTTCAACTACTACAAGAAGCACGGCTACAAC

ACCATTGTCATGGGTGCTTCCTTCCGTAACACTGGTGAAATCACCGAGCTTGCTGGCTGT

GACTACCTGACCATCTCCGTAAGTTTTGAGCGCCATTGGCTATGACTTGTCACAGTACTA

ACGGGATGCGTGTAGCCCAACCTCCTCGAGGATCTCTACAACTCTACCGCCGCTGTCCCC

AAGAAGCTCGACGCCGCCAGCGCTACCGGCCTTGACATCCCCAAGAAGACCTACATCAAT

GACGAGGCTCTGTTCCGCTTCGAGTTCAACGAGGAGGCCATGGCCGTCGAGAAGCTGCGT

GAGGGTATCTCCAAGTTCGCGGCCGATGCCGTGACCCTGAAGGACATCCTCAAGCAGAAG

GTCCAGGCCTAAAGGGAGACTGTCACTTGCATTAATTAAAGTTATGTCCATGCAATGTCC

TCATGTAAAAATTGGCGACCTACAAAATTGGTTTAGTAGATACGATTCATGAATGAGTTA

TCTTTCACTTTCTTTTTTTTTTTTCTTTTCCCCACATGTGGCTACATATTGCGGGCTGCC

TTATAGTATTGCTTGATCAGGGTTGTAATTGAATATGCAGCTGTAGGAAAGCAACCAGTG

GCAAGCTACAAGATGGAGACGGGACTTGGTGTCGATCTGGAGCTATCGGACCGGAAGCGG

GAACGAGCAGTTGTCAAGCTCGACTGCGTGCTTGAAGACAATCGATACAGGGCCTAGCCG

AGGCAAGCACGGGAGGAACTTGGTTTTAAAGCTGAGGAACATTCTTTCATCGGTTCTACT

CACCTAATAAGTGACAGTTGCAATTGGAAAATCTGCATGAACCGTAGCTTCCTCGGACTT

GGAGGCTGTGACACAGCTGGTCTGCATTAGTAGCACATCTTCCTGCATGGTGGGGTCGTA

CGGGTGGGTGGCTTTGTTGGGGCACCGGAACAAAGTAGAGCCACAGCTATTAGTAGTATT

TCGTGTCACTACAATCAATGTACAGGGAAGAAAGCAGAGGTAATGAATGGCTTCGCCAGG

GCTTGCGATCTGGACCGGCGATGCTTAGGAATCGCCGGAAGAGGGAAGTGACTCGTGCGG

GTGGGATTTTCATTTCATGCAAAAATACACCACAAGGACGTCATTCCCGAAAGACCCCGC

CAAGTGGGGGTGCGTTGCCCTTGGTTTCCCTCCGATTTTTCTTACTGTATGCTGTAATAT

CAATACCAGCGAGGGAAGAACTGGAAGCTGATCGAATCCGTTCTTGACTGGGGCCCTCAG

TGTACTCAGTGAGTTAGTACTGTATTAGTAGTACTGCTGCTTAAAGCTAGTCATGATGGA

ATCCCGTCATATTAGTTACCCCTCATCCCTCCATTTCCCCTTCTCTCTCTCCTCTCCTCT

TCTTTCTCCCTTCCATCTTC


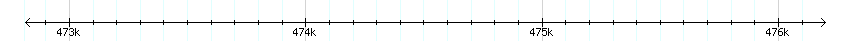


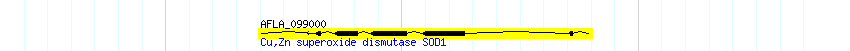


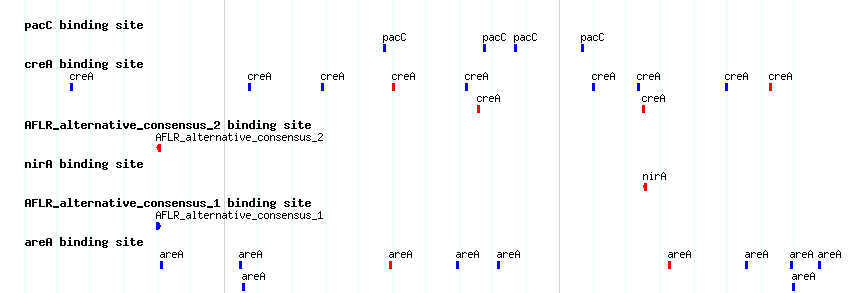


Program NSITE (Softberry Inc.) | Version 2.2004

Search for motifs of 8458 Regulatory Elements (REs) | SET of REs: RE SET from Ghosh DB

____________________________________________________________

Search PARAMETRS:

Expected Mean Number : 0.0100000

Statistical Siginicance Level : 0.9500000

Level of homology between known RE and motif: 80%

Variation of Distance between RE Blocks : 20%

NOTE: Mism. - Mismatches | Mean. Exp. Number - Mean Expected Number | Up.Conf.Int. - Upper Confidence Interval

============================================================

QUERY: >supercontig:JCVI-afl1-v2.0:EQ963484:473407:476791:-1

Length of Query Sequence: 2000 bp | Nucleotide Frequencies: A - 0.24 G - 0.24 T - 0.26 C - 0.26

............................................................

RE: > SiteID: S00154 | SiteName: EIIaE-T1 | FactorName: unknown | Org/Species/Gene: adenovirus EIIaE early | COMMENT: pseudo-TATA sequence required for constitutive EIIaE1 transcription | Ref: EMBO J 4: 1

Motifs on "-" Strand: Mean Exp. Number 0.00623 Up.Conf.Int. 1 Found 1

945 GTCCTTcAGgGTCA 932 (Mism.= 2)

............................................................

RE: > SiteID: S00239 | SiteName: LN4 | FactorName: unknown | Org/Species/Gene: M musculus Ig mu | Ref: EMBO J 5: 1791-7 (1986)

Motifs on "+" Strand: Mean Exp. Number 0.00384 Up.Conf.Int. 1 Found 1

957 GAAgGTCCAGGC 968 (Mism.= 1)

............................................................

RE: > SiteID: S00739 | SiteName: (TFIID/TBP)-H2B1 | FactorName: (TFIID/TBP) | Org/Species/Gene: histone H2B1 | Ref: Cell 50: 347-59 (1987)

Motifs on "+" Strand: Mean Exp. Number 0.00624 Up.Conf.Int. 1 Found 1

63 TATAAATAG 71 (Mism.= 0)

............................................................

RE: > SiteID: S01044 | SiteName: vaccinia-early-US | FactorName: unknown | Org/Species/Gene: VSV VGF | Ref: Proc Natl Acad Sci U S A 84: 6069-73 (1987)

Motifs on "+" Strand: Mean Exp. Number 0.00680 Up.Conf.Int. 1 Found 1

1875 TAgTACTGtATTAgTA 1890 (Mism.= 3)

............................................................

RE: > SiteID: S01124 | SiteName: HSE-hsp23 | FactorName: (HSTF) | Org/Species/Gene: hsp23 gene | COMMENT: derived from weak homology to hsp70 at identical position relative to TATA | Ref: Cell 30: 517-28

Motifs on "+" Strand: Mean Exp. Number 0.00604 Up.Conf.Int. 1 Found 1

888 CGAGAAGcTgCGTG 901 (Mism.= 2)

............................................................

RE: > SiteID: S01213 | SiteName: E2 RS.2 | FactorName: BPV-E2 | Org/Species/Gene: BPV genome | COMMENT: K-rel = 6.6 | Ref: Genes Dev 3: 510-26 (1989)

Motifs on "-" Strand: Mean Exp. Number 0.00896 Up.Conf.Int. 1 Found 1

461 GCACCGGCctCGGcAG 446 (Mism.= 3)

............................................................

RE: > SiteID: S01560 | SiteName: TIE (1) | FactorName: T3R | Org/Species/Gene: R norvegicus growth hormone | Ref: Mol Endocrinol 2: 536-42 (1988)

Motifs on "+" Strand: Mean Exp. Number 0.00550 Up.Conf.Int. 1 Found 1

59 AcaGTATAAAtAGGGC 74 (Mism.= 3)

............................................................

RE: > SiteID: S02589 | SiteName: Su3 | FactorName: unknown | Org/Species/Gene: M musculus Surf-1/Surf-2 | Ref: Mol Cell Biol 11: 1281-1294 (1991)

Motifs on "+" Strand: Mean Exp. Number 0.00919 Up.Conf.Int. 1 Found 1

614 gTGCTTCCTTCCGtaA 629 (Mism.= 3)

............................................................

RE: > SiteID: S02643 | SiteName: HB3 | FactorName: Hb | Org/Species/Gene: unknown | Ref: EMBO J 10: 2267-2278 (1991)

Motifs on "-" Strand: Mean Exp. Number 0.00627 Up.Conf.Int. 1 Found 2

1111 GgAAAAGAAAAA 1100 (Mism.= 1)

1105 GAAAAAaAAAAA 1094 (Mism.= 1)

............................................................

RE: > SiteID: S02910 | SiteName: G30 | FactorName: TBP | Org/Species/Gene: unknown | Ref: J Biol Chem 267: 20943-20952 (1992)

Motifs on "-" Strand: Mean Exp. Number 0.00289 Up.Conf.Int. 1 Found 1

1948 GGatGAGGGGTAAcTAA 1932 (Mism.= 3)

............................................................

RE: > SiteID: S02941 | SiteName: core promoter/TATA | FactorName: unknown | Org/Species/Gene: unknown | Ref: Genes Dev 5: 2225-2234 (1991)

Motifs on "+" Strand: Mean Exp. Number 0.00148 Up.Conf.Int. 1 Found 1

251 GATTAATATAGGtcT 265 (Mism.= 2)

............................................................

RE: > SiteID: S03168 | SiteName: MLC-2-site-S | FactorName: unknown | Org/Species/Gene: G gallus MLC-2 | Ref: Mol Cell Biol 12: 1107-1116 (1992)

Motifs on "-" Strand: Mean Exp. Number 0.00062 Up.Conf.Int. 1 Found 1

1810 GCTGGTaTtgaTATTACAGC 1791 (Mism.= 4)

............................................................

RE: > SiteID: S03290 | SiteName: apoE-B1-III | FactorName: unknown | Org/Species/Gene: H sapiens apolipoprotein E | Ref: J Biol Chem 263: 8300-8308 begin_of_the_skype_highlighting 8300-8308 end_of_the_skype_highlighting (1988)

Motifs on "+" Strand: Mean Exp. Number 0.00589 Up.Conf.Int. 1 Found 1

735 GCCCaACCTCCT 746 (Mism.= 1)

............................................................

RE: > SiteID: S03654 | SiteName: NFalpha4-T cell receptor alpha | FactorName: NFalpha4 | Org/Species/Gene: unknown | Ref: EMBO J 8: 729-733 (1989)

Motifs on "-" Strand: Mean Exp. Number 0.00435 Up.Conf.Int. 1 Found 1

168 tGGAGACCTTTC 157 (Mism.= 1)

............................................................

RE: > SiteID: S03838 | SiteName: f(alpha)-f(epsilon)-rpL30 | FactorName: f(alpha)-f(epsilon) | Org/Species/Gene: unknown | Ref: Genes Dev 3: 1789-1800 (1989)

Motifs on "-" Strand: Mean Exp. Number 0.00705 Up.Conf.Int. 1 Found 1

1259 CGCTTCCGG 1251 (Mism.= 0)

............................................................

RE: > SiteID: S03842 | SiteName: GABP-IE3 | FactorName: GABP | Org/Species/Gene: unknown | Ref: Science 253: 789-792 (1991)

Motifs on "+" Strand: Mean Exp. Number 0.00531 Up.Conf.Int. 1 Found 1

1248 GGAcCGGAAGCGGgAA 1263 (Mism.= 2)

............................................................

**RE: > SiteID: S03908 | SiteName: AP-1-NPY | FactorName: AP-1 | Org/Species/Gene: H sapiens neuropeptide Y | Ref: J Biol Chem 265: 12933-12939 (1990)**

**Motifs on "+" Strand: Mean Exp. Number 0.00650 Up.Conf.Int. 1 Found 1**

**515 aCAAGCGTGACTaC 528 (Mism.= 2)**

............................................................

RE: > SiteID: S04129 | SiteName: AGP-site A | FactorName: AGP/EBP | Org/Species/Gene: M musculus alpha 1-acid glycoprotein-1 | Ref: Mol Cell Biol 10: 6642-6653 (1990)

Motifs on "+" Strand: Mean Exp. Number 0.00746 Up.Conf.Int. 1 Found 1

1443 AGgcTGTGaCACAGCT 1458 (Mism.= 3)

Motifs on "-" Strand: Mean Exp. Number 0.00746 Up.Conf.Int. 1 Found 1

1365 AGAaTGTtCCtCAGCT 1350 (Mism.= 3)

............................................................

RE: > SiteID: S04451 | SiteName: opaque-2-albumin b-32_(3) | FactorName: opaque-2 | Org/Species/Gene: Z mays albumin b-32 | Ref: EMBO J 10: 617-624 (1991)

Motifs on "-" Strand: Mean Exp. Number 0.00214 Up.Conf.Int. 1 Found 1

233 GATGATGTGG 224 (Mism.= 0)

............................................................

RE: > SiteID: S04852 | SiteName: Sp1-OMP_(3) | FactorName: Sp1 | Org/Species/Gene: R norvegicus OMP | Ref: Mol Cell Biol 13: 3002-3014 begin_of_the_skype_highlighting 3002-3014 end_of_the_skype_highlighting (1993)

Motifs on "-" Strand: Mean Exp. Number 0.00975 Up.Conf.Int. 1 Found 1

193 AAAGaGAGGCGaGCaT 178 (Mism.= 3)

............................................................

RE: > SiteID: S05361 | SiteName: NF-Y-Cyc1A-2 | FactorName: NF-Y | Org/Species/Gene: unknown | COMMENT: secondary source db: NF-Y resource | Ref: Nucleic Acids Res 26: 1135-43 (1998)

Motifs on "-" Strand: Mean Exp. Number 0.00883 Up.Conf.Int. 1 Found 1

1378 TAGaACCgATGAAAGa 1363 (Mism.= 3)

............................................................

RE: > SiteID: S05422 | SiteName: ROX3 regulatory element | FactorName: undefined | Org/Species/Gene: unknown | Ref: Genetics 142: 1083-93 (1996)

Motifs on "-" Strand: Mean Exp. Number 0.00278 Up.Conf.Int. 1 Found 2

1104 aAAAAAAAAAAaGAA 1090 (Mism.= 2)

1103 aAAAAAAAAAAGaAA 1089 (Mism.= 2)

............................................................

RE: > SiteID: S05773 | SiteName: AP-2-VGF (2) | FactorName: AP-2 | Org/Species/Gene: H sapiens VGF | COMMENT: secondary source db gi:gb|2414517 may not be verified experimentally | Ref: J Neurochem 68: 13

Motifs on "-" Strand: Mean Exp. Number 0.00581 Up.Conf.Int. 1 Found 1

980 GTCTCCCTT 972 (Mism.= 0)

............................................................

RE: > SiteID: S06067 | SiteName: HIOMT-B-E1 | FactorName: CRX | Org/Species/Gene: R norvegicus HIOMT | Ref: Proc Natl Acad Sci USA 95: 1876-81 (1998)

Motifs on "-" Strand: Mean Exp. Number 0.00060 Up.Conf.Int. 1 Found 1

1426 TACGgTTcATGCAgATTT 1409 (Mism.= 3)

............................................................

RE: > SiteID: S06075 | SiteName: c-Myb-c-Erb-B2 site 5 | FactorName: c-Myb | Org/Species/Gene: H sapiens c-Erb-B2 | Ref: J Biol Chem 270: 9384-9 (1995)

Motifs on "-" Strand: Mean Exp. Number 0.00681 Up.Conf.Int. 1 Found 1

555 CTTAACACC 547 (Mism.= 0)

............................................................

RE: > SiteID: S06395 | SiteName: pMT-motif-12 | FactorName: undefined | Org/Species/Gene: (Pike) MT-I/II | Ref: Genome Res 12: 739-48 (2002)

Motifs on "-" Strand: Mean Exp. Number 0.00748 Up.Conf.Int. 1 Found 1

1352 GCTTTAAAA 1344 (Mism.= 0)

............................................................

RE: > SiteID: S06678 | SiteName: Brn-3-CRH | FactorName: Brn-3 | Org/Species/Gene: R norvegicus CRH | Ref: Proc Natl Acad Sci USA 93: 15097-101 (1996)

Motifs on "-" Strand: Mean Exp. Number 0.00164 Up.Conf.Int. 1 Found 1

1179 TGCATAtTcAATTAC 1165 (Mism.= 2)

............................................................

RE: > SiteID: S06884 | SiteName: Egr-1-alpha-MHC | FactorName: Egr-1 | Org/Species/Gene: R norvegicus alpha-myosin heavy chain | Ref: J Biol Chem 266: 12813-6 (1991)

Motifs on "+" Strand: Mean Exp. Number 0.00683 Up.Conf.Int. 1 Found 1

1744 GTGGGGGTG 1752 (Mism.= 0)

............................................................

RE: > SiteID: S07079 | SiteName: PU.1-M-CSF | FactorName: PU.1 | Org/Species/Gene: H sapiens M-CSF | Ref: Mol Cell Biol 14: 373-81 (1994)

Motifs on "-" Strand: Mean Exp. Number 0.00985 Up.Conf.Int. 1 Found 1

1985 AAAGAaGaGGAGAG 1972 (Mism.= 2)

............................................................

RE: > SiteID: S07713 | SiteName: Spi-1-Iglambda-2-4 | FactorName: Spi-1 | Org/Species/Gene: unknown | Ref: Oncogene 11: 303-13 (1995)

Motifs on "-" Strand: Mean Exp. Number 0.00100 Up.Conf.Int. 1 Found 1

1100 AAAaAAAAGaAAGTGAAAga 1081 (Mism.= 4)

............................................................

RE: > SiteID: S07895 | SiteName: Xbp1-CYS3 | FactorName: Xbp1 | Org/Species/Gene: unknown | Ref: Mol Cell Biol 20: 478-87 (2000)

Motifs on "+" Strand: Mean Exp. Number 0.00470 Up.Conf.Int. 1 Found 1

743 TCCTCGAGGATc 754 (Mism.= 1)

............................................................

RE: > SiteID: S08160 | SiteName: YY1-Ig-kappa3_-enhancer | FactorName: YY1 | Org/Species/Gene: Igkappa | Ref: Proc Natl Acad Sci USA 88: 9804-8 (1991)

Motifs on "-" Strand: Mean Exp. Number 0.00575 Up.Conf.Int. 1 Found 1

1218 CTCCATCTT 1210 (Mism.= 0)

............................................................

RE: > SiteID: S08299 | SiteName: Nmp4-COL1A1-site-A | FactorName: Nmp4 | Org/Species/Gene: R norvegicus COL1A1 | Ref: J Biol Chem 277: 16153-9 (2002)

Motifs on "+" Strand: Mean Exp. Number 0.00124 Up.Conf.Int. 1 Found 2

1090 TTCTTTTTTTTTtTTcTTtT 1109 (Mism.= 3)

1093 TTtTTTTTTTTTCTTTTcCc 1112 (Mism.= 3)

............................................................

RE: > SiteID: S08300 | SiteName: Nmp4-COL1A1-site-B | FactorName: Nmp4 | Org/Species/Gene: R norvegicus COL1A1 | Ref: J Biol Chem 277: 16153-9 (2002)

Motifs on "+" Strand: Mean Exp. Number 0.00111 Up.Conf.Int. 1 Found 1

1093 TTtTTTTTTTTTCTTTtCCc 1112 (Mism.= 3)

............................................................

Totally 38 motifs of 34 different REs have been found
